# Rare Earth-Enhanced Laser Cladding Metal-Based Coatings: A Review

**DOI:** 10.3390/ma19163504

**Published:** 2026-08-18

**Authors:** Jingwei Xiao, Dongbo Tao, Yangyang Zheng, Jingqin Yang, Longxiao Huang, Wei Liu, Hanguang Fu, Yulong Li, Kaiming Wang

**Affiliations:** 1 College of Mechanical and Vehicle Engineering, Changsha University of Science and Technology, Changsha 410114, China18973688333@163.com (J.Y.); 2 College of Mechanical Engineering, Hunan Mechanical & Electrical Polytechnic, Changsha 410151, China; 3 Beijing Institute of Control Engineering, Beijing 100190, China; 18518134081@163.com; 4 College of Materials Science and Engineering, Beijing University of Technology, Beijing 100124, China; hlx2022@emails.bjut.edu.cn (L.H.); lw1538251436@163.com (W.L.); hgfu@bjut.edu.cn (H.F.)

**Keywords:** laser cladding, rare earth elements, microstructure, wear resistance, corrosion resistance, microhardness

## Abstract

Laser cladding technology is a widely applied surface modification technique; but its inherent process characteristics render it susceptible to cracking. The addition of rare earth oxides has proven to be an effective approach for curbing crack formation and enhancing the comprehensive performance of the coating. This review summarizes the mechanisms by which rare earth additives improve the coating microstructure, molten bath behavior, and interfacial bonding strength, including adjusting surface tension, purifying the molten bath, and forming interatomic chemical bonding. The addition of rare earth oxides significantly improves the forming quality of materials, which contributes to a finer and more uniform microstructure and directly enhances material hardness and resistance to plastic deformation, thereby altering wear behavior and improving wear resistance. The increased hardness provides better support for the surface oxide film, while the improved microstructure mitigates galvanic corrosion and intergranular corrosion susceptibility, leading to enhanced corrosion resistance. In addition, the article incorporates relevant quantitative analysis to provide a reference basis for the type selection, content optimization, and particle size selection of rare earth additives. This article provides a coherent framework for understanding how the addition of rare earths transfers its effects from the process to the performance. However, the industrial application of rare earth oxide laser cladding faces key bottlenecks such as additive deactivation under extreme conditions, threshold effects, nano-agglomeration, and cost constraints.

## 1. Introduction

During service, mechanical components are always subjected to wear and fatigue damage induced by cyclic loading, stress concentration, and other mechanical effects. In addition, long-term exposure to high-temperature environment will bring creep effects. The corrosive media can also induce various types of environmental damage [1,2,3,4,5,6],which pose severe challenges to the service process of engineering metallic materials. However, surface technologies such as thermal spraying, surface welding, and laser cladding can repair the surface of damaged metal components, then significantly improving their long-term durability, wear resistance, and mechanical properties [7,8,9,10,11,12]. As a result, they are widely applied in key fields such as aerospace, marine engineering, and remanufacturing of engineering machinery.

Among them, laser cladding technology has been widely promoted due to its outstanding advantages such as low heat input and dilution rate, where its interfacial bonding strength is also far superior to traditional processes. Its basic principle is delivering specific alloy powder materials to the substrate surface by pre-placed or synchronous powder feeding methods. Then, high-energy laser irradiation will rapidly melt the powder material and a thin layer of the substrate surface to form a molten bath. Subsequently, it will be rapidly solidified, producing a cladding layer that possesses a low dilution rate and exhibits a metallurgical bond with the substrate [13], ultimately achieving performance enhancement, dimensional restoration, or functional recovery of the substrate [14,15,16]. Moreover, the extremely low dilution rate and minimal heat-affected zone minimized workpiece deformation to the greatest extent. Meanwhile, as an environmentally friendly process, it avoids the environmental pollution issues caused by traditional methods. In addition, its wide range of material selection and the capability for selective cladding bring about an excellent performance-to-price ratio.

Although laser cladding offers vital advantages, its inherent process characteristics make it prone to defects such as cracks, pores, and residual stress during processing. This is owing to the differences in thermophysical properties, such as the shrinkage rate between cladding layer and substrate which can give rise to thermal stress and phase transformation stress. These two stresses will generate microscopic strain, and produce crack initiation from this location [17,18,19]. In the meantime, the rapid heating–cooling process produces a large temperature gradient and leads to significant accumulation of thermal stress, providing the internal driving force for crack initiation and propagation.

Furthermore, it is widely recognized that laser power, scanning speed, and powder feed rate directly influence the microstructure and properties of the coating [20,21]. Specifically, higher laser power can increase the temperature and fluidity of the molten bath. Consequently, the uniformity of the coating is improved. However, excessively high power will reduce the cooling rate, leading to coarser grains and decreased hardness. On the other hand, the faster scanning speed shortens laser interaction time, bringing about reduced heat input, narrower coating width, and lower coating height. It also tends to disrupt the equilibrium of the molten bath, causing uneven edges. The powder feed rate is associated with the powder deposition amount per unit volume. An appropriately increased powder feed rate can increase the energy absorbed per unit volume of powder, providing a more uniform coating structure and higher hardness. In contrast, if the powder feed rate progressively increases, the coating uniformity will first increase and then decrease, which can even cause defects [22,23].

To address the abovementioned issues, researchers have proposed some corresponding countermeasures such as introducing a ductile transition layer between the cladding layer and the substrate, preheating the substrate in combination with process parameter optimization [17], and developing novel powder systems [18], in which the addition of a transition layer can coordinate strain, but it may lead to drawbacks such as thermal expansion mismatch, reduced strength, and increased corrosion risk, and will become more obvious under high-temperature, corrosive or irradiation environments. The substrate preheating method often gives rise to elemental dilution, which needs to establish a highly correlated powder system to counteract the negative impacts generated during the pretreatment process. In this context, the application of rare earth oxides has garnered widespread attention in recent research.

Rare earth elements’ unique characteristics significantly improved the coating performance. Specifically, the majority of rare earth oxide crystals exhibits a fluorite or hexagonal structure that is highly compatible with common matrix structures such as Fe and Ni, possessing low lattice mismatch. Therefore, they can serve as heterogeneous nucleation substrates to promote nucleation [24]. Moreover the 4f electron characteristics facilitate rare earth atoms to undergo charge transfer at the interface, forming charge dipole layer [25]. Its high surface activity significantly reduces the surface tension of the molten metal, i.e., the interfacial energy γSL. According to classical nucleation theory (Equations (1) and (2)) and Young’s equation (Equation (3)), this change drastically reduces the nucleation activation energy ΔG*. Then, the nucleation rate increases; the critical crystal nucleus size decreases, and more crystal embryos reach a stable state. It also reduces the contact angle θ during that time, and the spreading ability of the melt on the substrate surface and the wettability of molten bath are improved [26,27].

(1) $$\Delta G^{*}=\frac{3\Delta G_{v}^{2}}{16\pi \gamma_{SL}^{3}}$$

(2) $$\Delta G^{*}=\frac{16\pi \gamma^{3}}{3\Delta G_{v}^{2}}$$

(3) $$\cos\theta =\frac{\gamma_{sv}-\gamma_{sl}}{\gamma_{lv}}$$

Rare earth additives can also enhance the durability of components under prolonged wear, high-temperature service, and corrosive environments. First, owing to their high surface activity, rare earth elements can lower the nucleation energy barrier and serve as heterogeneous nucleation sites to promote nucleation. They also react with impurities in the molten bath, facilitating their removal to achieve melt purification, while simultaneously modifying the morphology of the remaining inclusions. This process directly eliminates the sources of crack initiation from within, reduces elemental segregation and internal porosity defects, and yields a more uniform and denser microstructure, thereby extending the fatigue life of materials under high-temperature service conditions. Furthermore, the resulting grain refinement enhances the hardness and resistance to plastic deformation. Combined with the solid solution strengthening, pinning effect, and dispersion strengthening induced by the rare earth additives, crack propagation is effectively impeded. Consequently, the forming quality of the material is improved, and the risk of surface spalling is reduced, thereby prolonging the service life of components under long-term wear and external loading conditions. In addition, this refinement also suppresses the initiation of galvanic corrosion and reduces the pathways for corrosive media penetration. The high chemical activity of rare earth elements also lowers the formation energy of oxides, promoting the development of a stable passive film on the surface, which hinders the diffusion of corrosive species and enhances the durability of components in corrosive environments.

In recent years, multiple review articles have addressed the application of rare earth elements in laser cladding. Some review articles only cover the effect of a single additive on a single alloy coating, or are limited to the study of a single property. This review systematically concluded the modification mechanisms and performance improvements of three rare earth oxides (CeO_2_, La_2_O_3_, and Y_2_O_3_) in Fe-based, Ni-based, Co-based, and high-entropy alloy coatings. A complete performance transfer chain spanning forming quality, microhardness, wear resistance, and corrosion resistance is constructed, revealing the intrinsic logic from microstructure optimization to macroscopic service performance enabled by rare earth modification. Quantitative references are provided for the type of selection, content optimization (0.5–2.0 wt.%), and particle size selection of rare earth additives. Furthermore, the differentiated characteristics of each rare earth element are clearly identified, along with the existing practical bottlenecks and future breakthrough directions in industrial applications.

## 2. Action Mechanisms of Rare Earth Elements in Laser Cladding

### 2.1. Microstructural Refinement Mechanism

#### 2.1.1. Grain Refining

**Figure 1:**
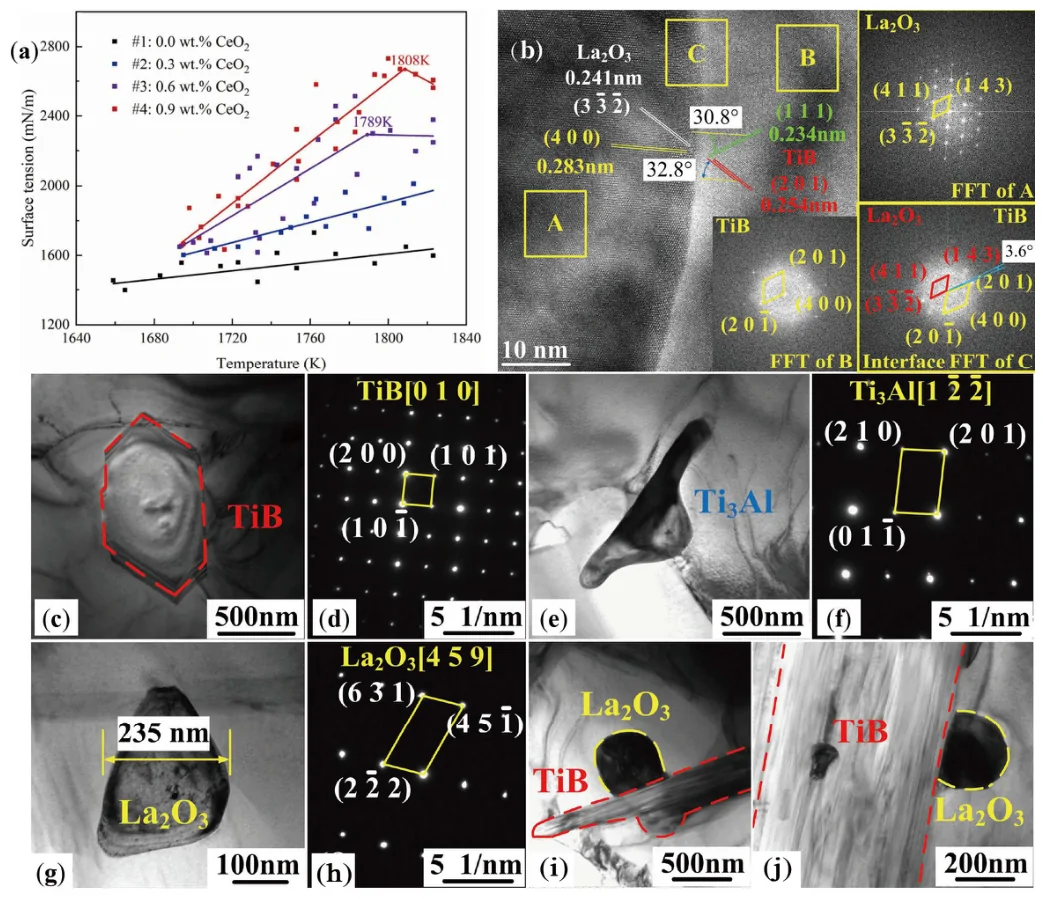
(**a**) Temperature dependence of the surface tension of the alloy powder [28]. (**b**) HRTEM image of the TiB/La_2_O_3_ interface and the corresponding FFT patterns. Region A corresponds to the TiB area, region B to the La_2_O_3_ area, and region C to the TiB/La_2_O_3_interface region. (**c**–**j**) Bright field TEM images of the reinforcing particles and corresponding selected-area electron diffraction patterns [29].

Rare earth oxides can diminish the nucleation activation energy by reducing the surface tension and serve as heterogeneous nucleation substrates to achieve grain refinement. Lichao Liu [28] et al. developed T15 high-speed steel coatings with different contents of nano-CeO_2_. Because Ce can alter the positive correlation between surface tension and temperature via desulfurization and deoxygenation, the nucleation is being promoted. As shown in Figure 1a, O and S elements easily tend to adsorb onto the molten bath surface, reducing the surface tension. Upon the temperature increases, these elements are desorbed, and part of the surface tension is restored, resulting in a positive temperature coefficient. However, when the CeO_2_ content is over 0.6 wt.%, O and S are largely consumed. The surface tension released by desorption is insufficient to offset the decline caused by the temperature rise; the temperature coefficient turns negative, and the nucleation energy barrier is reduced, thereby promoting nucleation, increasing the nucleation rate and refining the grains. Yueqiao Feng [29] et al. in situ synthesized La_2_O_3_ on the (Ti_3_Al + TiB)/Ti composite. As shown in Figure 1b–j, the La_2_O_3_ particles are closely adjacent to the TiB phase, and their lattice mismatch only by 4.72%. Moreover, there is a parallel orientation relationship existing between (201) TiB and (332) La_2_O_3_. This indicates that La_2_O_3_ can provide an effective interface for its nucleation to refine the grains. It enables more grains to nucleate simultaneously, and their growth is restricted, resulting in the refinement of grain size and a tendency towards uniformity. The specific data on the change in grain size are shown in Table 1. Thus, the grain structure of the coating becomes finer, which improves the yield strength of the material through the Hall–Petch effect (Equation (4)), thereby enhancing the stability of the macroscopic mechanical properties. The $\sigma_{y}$ is the yield strength; $\sigma_{0}\;$ is the friction stress (lattice resistance to dislocation motion); k is the Hall–Petch coefficient (a measure of grain boundary strengthening), and d is the average grain diameter.

(4) $$\sigma_{y}=\sigma_{0}+\frac{k}{\sqrt{d}}$$

These studies demonstrate that rare earth oxides can modulate the surface tension of metals due to their high surface activity, then reduce the nucleation energy barrier and promote nucleation. Meanwhile, their unique crystal structure exhibits a low lattice mismatch with the majority of materials, making them serve as heterogeneous nucleation substrates to achieve grain refinement.

#### 2.1.2. Suppression of Columnar Grains Growth

**Figure 2:**
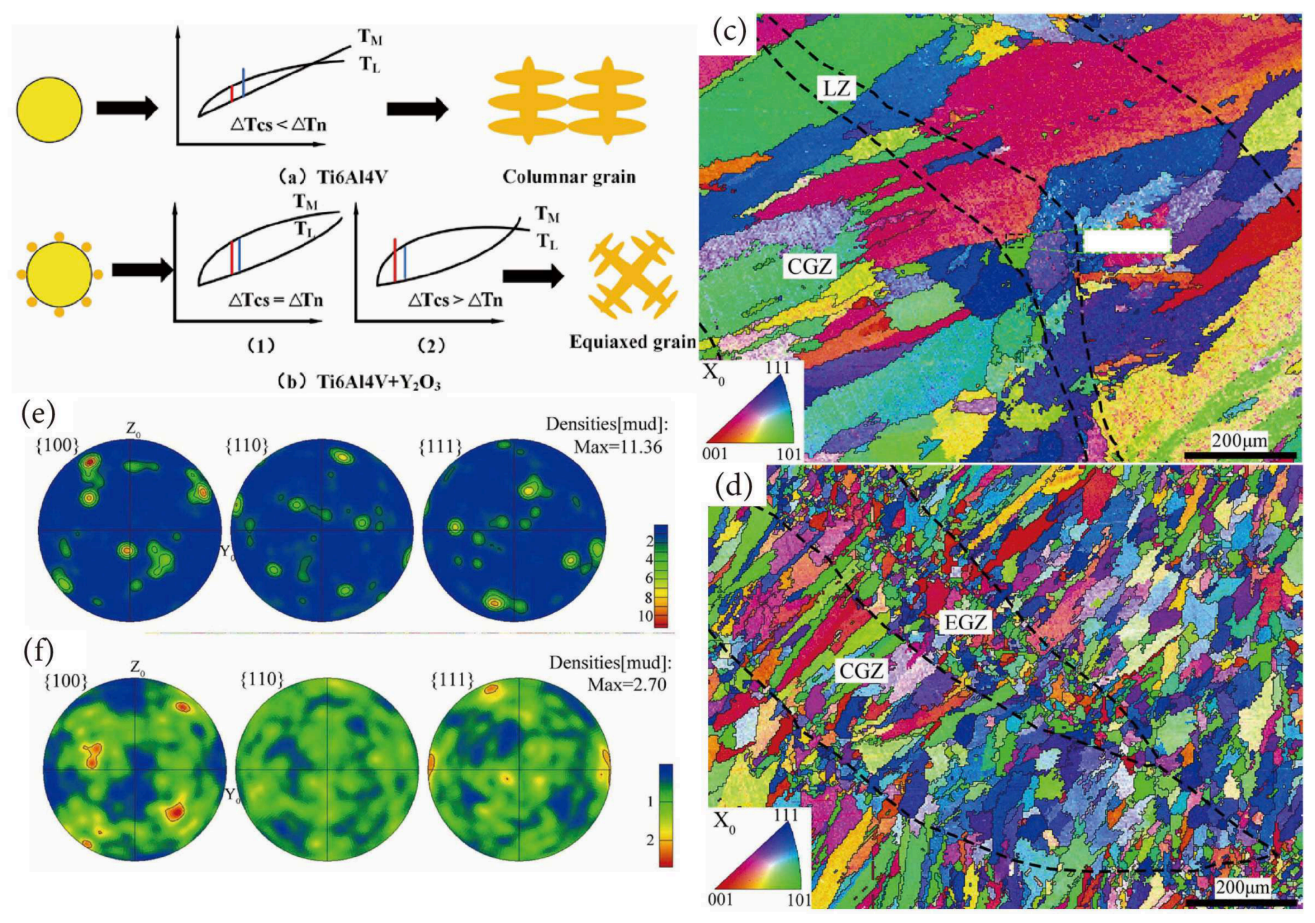
(**a**,**b**) Schematic diagrams of the grain equiaxialization mechanism of Ti6Al4V-xY_2_O_3_. (**a**) Ti6Al4V, (**b**) Ti6Al4V-xY_2_O_3_. TM—actual temperature distribution of the melt; TL—equilibrium liquidus temperature distribution. The red and blue lines represent ΔTcs and ΔTn, respectively [31]; inverse pole figure (IPF)-colored maps and pole figures of the transverse section (YZ) at the top layer: (**c**,**e**) Ni625 cladding layer; (**d**,**f**) Ni625 + 0.2 wt.%CeO_2_ cladding layer [32].

The rapid heating and cooling characteristics of the laser cladding process cause the columnar grains to grow epitaxially, adversely affecting the microstructural uniformity and properties of coatings [30]. Rare earth elements can increase the nucleation rate and facilitate the formation of equiaxed grain structures, effectively inhibiting the epitaxial growth of columnar grains. Wei Han [31] et al. introduced Y_2_O_3_ particles into the Ti6Al4V alloy matrix, and the decomposed Y element enriched at the solid–liquid interface front, forming a constitutional undercooling zone (Figure 2a,b). This drives Y_2_O_3_ particles to transform into heterogeneous nucleation substrates, then serving as sites for the surrounding phases to attach and grow. These sites can promote grain refinement, causing the grain structure to change from coarse columnar crystals to fine and uniform equiaxed crystals. This transformation reduces the risk of defects such as pores and cracks occurring at the coarse columnar crystals, which act as stress concentration points during the service process. Lisheng Zhan et al. [32] found that the Ni625 coating displays a strong crystallographic orientation in regularity. On the contrary, CeO_2_ promotes heterogeneous nucleation to realize grain refinement and renders the grain orientation to be more random, which is shown as the reduction in maximum pole density in the {100} pole figure (Figure 2c–f). This accelerates the cladding layer to transform from coarse columnar grains to a mixed structure of fine equiaxed grains and columnar grains, and suppresses the adverse effects of the columnar crystal structure on the microstructure uniformity and properties of the coating.

These phenomena are attributed to the large atomic radius of rare earth elements, which results in their low solubility in the solid phase. For this reason they are rejected by the solid–liquid interface during solidification and formation, thereby forming segregation at the dendrite tips which lowers the local liquidus temperature and induces constitutional undercooling that facilitates the growth of dendritic and other microstructures. When the degree meets the critical value required for nucleation, the rare earth particles are activated to provide effective nucleation substrates, bringing a more random crystallographic orientation, effectively suppressing the growth of planar interfaces and promoting the columnar-to-equiaxed transition [33].

### 2.2. Purification and Improvement of Metallurgical Bonding

#### 2.2.1. Purification Effect of Molten Bath

Surface-active elements such as O, S, and H in the molten bath typically exist as impurities and tend to adsorb onto the surface, influencing the flow behavior, morphological stability, and formation of structural defects in the molten bath by partially suppressing the surface tension [34], whereas rare earth atoms can react with these elements to form stable, high-melting-point compounds. Part of the products float to the surface and form removable slag; in this way melt purification is achieved [35,36]. The residual inclusions are dispersed throughout the coating.

**Figure 3:**
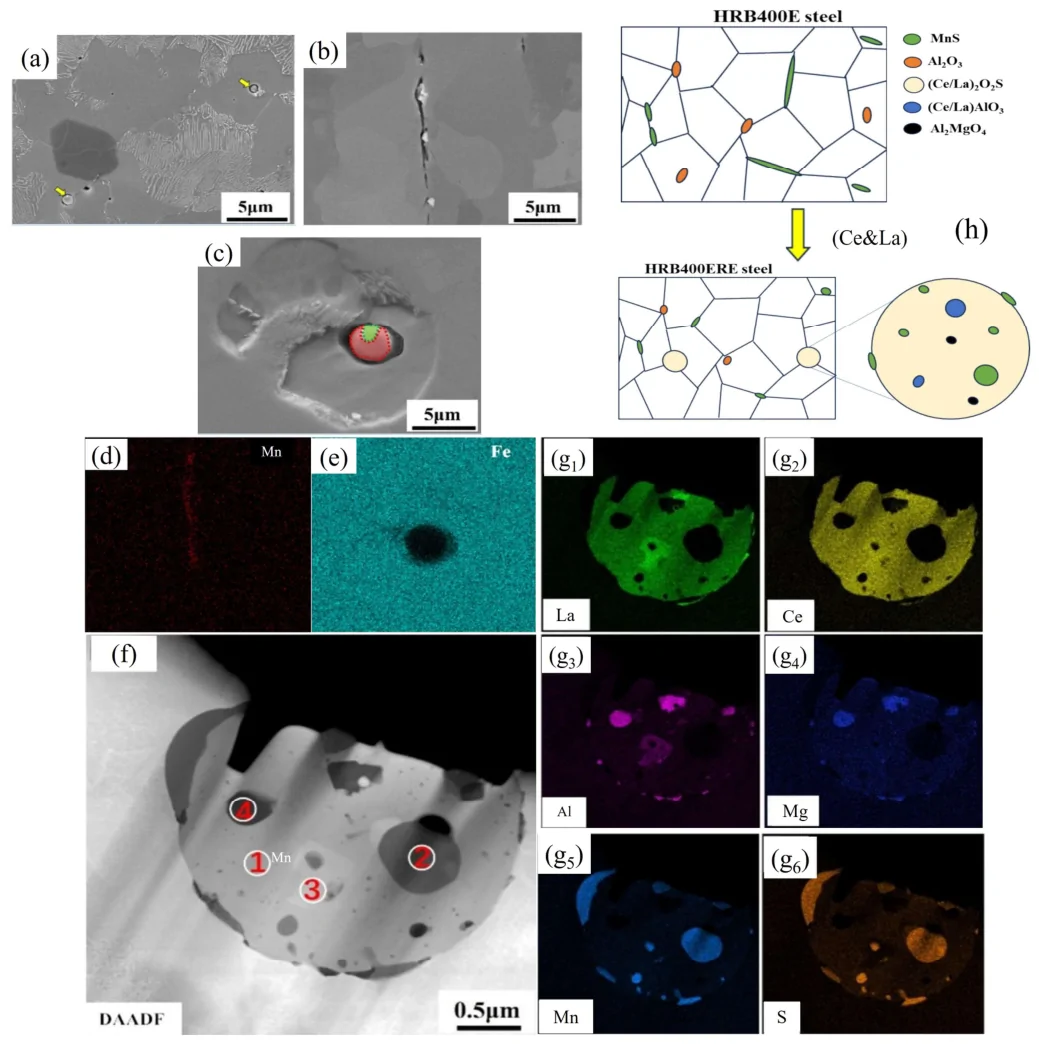
EDS analysis of the precipitates in the two steels: (**a**,**b**) the B steel, and (**c**–**e**) the B-RE steel. (**d**,**e**) Elemental distribution maps; TEM and EDS as results of the precipitates in the B-RE steel: (**f**) bright field image of the FIB sample, (**g1**–**g6**) mapping of element distribution [37], (**h**) Schematic of the microstructural evolution due to the addition of rare earths.

Qiang Yao [37] et al. investigated impurities, such as MnS, which are prone to form during the laser cladding process by adding La and Ce to the HRB400E low-alloy steel matrix. The elongated and irregularly shaped MnS and other inclusions are shown in Figure 3a,d, which originally served as preferential initiation sites for corrosion and have been refined and spheroidized into spherical or spindle-shaped forms, as shown in Figure 3c–e. This morphological transformation fundamentally eliminates the crack initiation sources, which is critical for improving the toughness and fatigue strength [38]. Furthermore, owing to the strong chemical bonding affinity for sulfur and oxygen, the CeO_2_ undergoes a displacement reaction with the components in inclusions, forming more thermodynamically stable (Ce/La)_2_O_2_S and (Ce/La)AlO_3_ particles (Figure 3h), and improving the toughness, fatigue strength, and corrosion resistance of the material. Additionally, the EDS mapping analysis of the precipitates in the B-RE steel (Figure 3(g1–g6)) revealed that the smaller-sized precipitates were enveloped by larger stable (Ce/La)_2_O_2_S phases.

The purification effect originates from the reactions of rare earth elements with elements such as O, S, and H, which reduce the viscosity of the molten bath and minimize defect formation. Meanwhile, the quantity and morphology of the remaining inclusions are optimized, with the result of their detrimental effects on material properties being mitigated.

#### 2.2.2. Enhancing the Metallurgical Bonding

**Figure 4:**
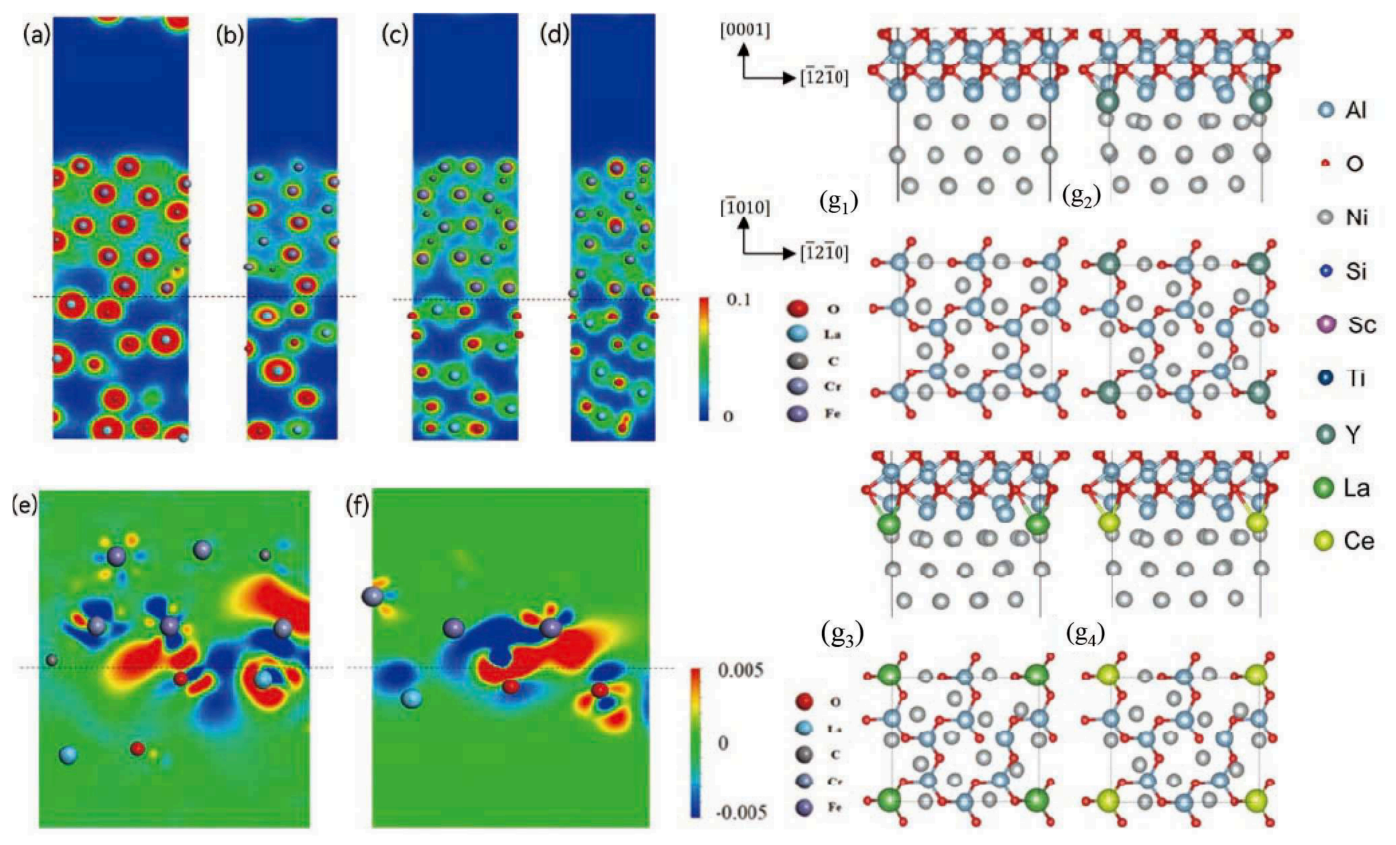
Differential charge density of La_2_O_3_ (111)//M7C3(0001) interface. La-M7C interface of (**a**) the front view; (**b**) the side view; O1-M7C interface of (**c**) the front view; (**d**) the side view; (**e**) La-M7C3; (**f**) O1-M7C3 [42]. (**g1**–**g4**) reflect the optimized structures of the initial interface, Y-doped coating, la-doped coating, and Ce-doped coating for α−Al_2_O_3_/γ−Ni successively [43].

In addition to reducing interfacial defects by the purification effect, rare earth elements also decrease the interfacial energy, accumulating atomically at the interface and forming covalent bonds with other atoms, which significantly improve the interfacial bonding strength [39,40]. Wenwei Song et al. [41] and Yuenian He et al. [42] conducted some interfacial analysis: the results revel that an interface between rare earth oxides and hard phases exhibits the highest work of adhesion and the lowest interfacial energy. As shown in Figure 4a–d, the charge density is continuously distributed across this interface, with charge accumulation and depletion. This indicates the formation of chemical bonding between atoms, leading to an improvement of interfacial stability. However, when La_2_O_3_ is in excess, they cannot fully form an ideal interface with M_7_C_3_; the remaining part will produce an agglomeration, disrupting the continuity and bonding quality of the interface. Negami et al. [43] added rare earth elements such as Y and Hf into MCrAlX coatings; the results revealed that RE elements enrich at the interface between the thermally grown oxide (TGO) and the γ-Ni metallic bond coat. They reduce the interfacial energy between α-Al_2_O_3_(0001) and γ-Ni(111), and form covalent bonds with Ni atoms. This enhances the bonding strength and adhesion at the α-Al_2_O_3_/metal substrate interface, inhibiting the spallation and multi-layering of the TGO and delaying coating failure accordingly. Furthermore, the RE atoms only caused minor local atomic position adjustments (Figure 4(g1–g4); this can rule out the possibility of rare earth oxides enhancing interfacial bonding by large-scale structural distortion.

The abovementioned research indicates that rare earth oxides can form a relatively stable heterogeneous nucleation interface with certain substances and generate charge transfer to form chemical bonding. It also enhances the energy absorption rate and promotes the melting of the substrate and elemental diffusion. As a result, the melt depth and dilution rate increase; a strong metallurgical bonding appears at the interface [44].

### 2.3. Enhance the Fluidity of the Molten Bath

**Figure 5:**
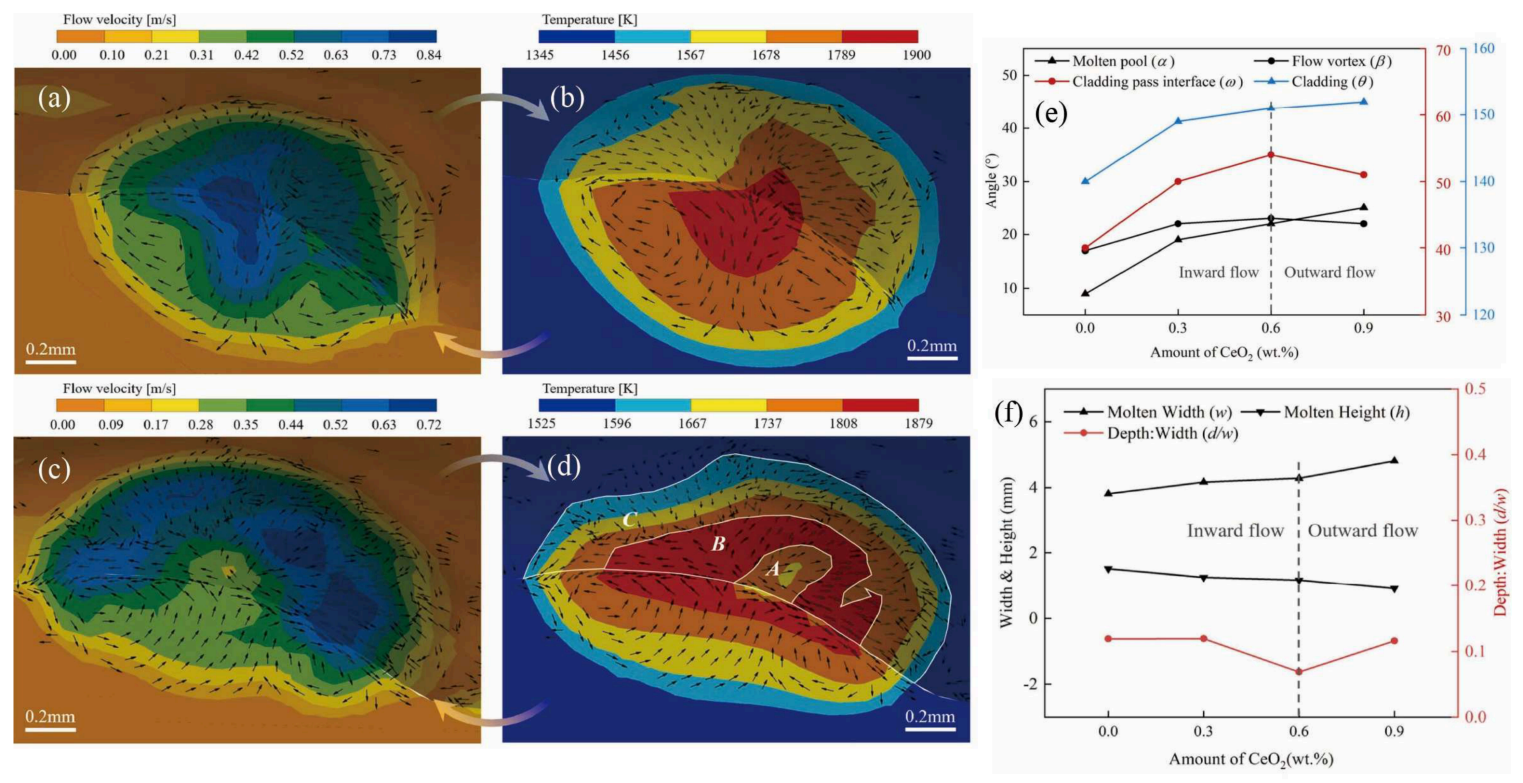
(**a**) Molten bath of T15 high-speed steel without CeO_2_, (**b**) molten bath with 0.3 wt.% CeO_2_, (**c**) molten bath with 0.6 wt.% CeO_2_, (**d**) molten bath with 0.9 wt.% CeO_2_; (**e**,**f**) are the variations in molten bath inclination and related dimensional parameters induced by CeO_2_ content, respectively [28].

The fluidity of the molten bath is primarily driven by the surface tension gradient, i.e., Marangoni convection, which directly determines the surface flatness and internal densification of the cladding layer. Rare earth oxides can adjust this effect to improve the molten bath behavior and geometry by enhancing the fluidity of the molten bath and the overlapping effect of laser cladding. Lichao Liu et al. [28] indicated that CeO_2_ can adjust the Marangoni flow by reducing the surface tension. As shown in Figure 5a–d, when CeO_2_ < 0.6 wt.% and the ∂γ/∂T > 0, surface tension drives the molten bath to flow from the high-temperature central toward the edges, forming a deeper wedge-shaped molten bath. When the content exceeds 0.6 wt.% and ∂γ/∂T becomes negative, a dual-flow pattern is presented with outward flow from the center and inward flow from the periphery, giving rise to a wider and shallower molten bath with enhanced fluidity. Moreover, as shown in Figure 5e,f, as CeO_2_ content increases from 0wt.% to 0.9 wt.%, the inclination angle of the molten bath (α) increases from 9° to 25°, which increases the inclination angle (ω) of the interpass interface and makes the lateral overlap more sufficient. The decreases in depth-to-width ratio (d/w) of the cladding layer enable the fusion line at the substrate to become flatter, improving the forming stability. However, it can also be clearly observed that when the CeO_2_ content increases to 0.9 wt.%, the ratio (d/w) and molten depth (d) rebound, indicating that an excessively high CeO_2_ content will weaken the optimization effect on the interface morphology.

The above effects, together with the purification effect and the stirring effect resulting from the density difference between them and the substrate [45], collectively improve the viscosity of the molten bath, achieving a uniform distribution of elements within the coating. The flow resistance caused by elemental segregation reduces, so the overall fluidity is enhanced. As a result, the efficiency of gas and impurity removal increases; the purification degree of the molten bath continues to improve, and the viscosity keeps rising, ultimately forming a positive cycle that continuously enhances the fluidity of the molten bath. But excessive rare earth elements generate excess impurities that are difficult to remove, reducing molten bath fluidity.

### 2.4. Strengthening Mechanism

#### 2.4.1. Solid Solution Strengthening

Under the rapid solidification condition in laser cladding, rare earth elements become supersaturated in the molten bath due to their large atomic radius, and dissolve into the crystal lattice of the substrate metal. This reduces the chemical potential gradient and interfacial energy at grain boundaries. Meanwhile, it also causes severe lattice distortion, generating an internal stress field that impedes dislocation motion, producing a solid solution strengthening effect promoting grain growth, and ultimately enhancing the strength and hardness of the material. Zhicheng Liu [46] et al. prepared WTaNbMo refractory high-entropy alloy coatings containing Y_2_O_3_ particles on an Inconel 718 substrate using laser cladding technology. In the WTaNbMo-Y_2_O_3_ coating, Y_2_O_3_ particles preferentially dissolved into the BCC solid solution, replacing the original solid solution atoms. Due to the larger ionic radius of Y^3+^ as compared to the atomic radii of the main RHEA elements (W, Ta, Nb, Mo), an atomic size mismatch occurred, leading to significant lattice expansion and severe lattice distortion. This distortion increases the internal micro-stress within the alloy, enabling the coating to better resist deformation during friction, thereby reducing the friction coefficient. Simultaneously, this distortion impedes dislocation motion and elevates the internal energy and micro-stress of the material, thereby enhancing the overall strength and wear resistance of the coating. It also indirectly improves the coating’s ability to resist the penetration of corrosive media [47,48]. Furthermore, solid solution strengthening is often accompanied by grain refinement. According to the relevant relationship in Equation (5), where *a* is a constant representing the corrosion rate at infinite dilution of the diffusing species, *b* is a constant related to the kinetics of the corrosion process, and *φ* is the flux of the corrosive species, the corrosion rate is inversely proportional to the average grain size. The fine-grained structure provides more active sites, promoting the formation of a more stable and denser passive film. This passive film effectively blocks the penetration of aggressive ions such as chloride ions, thereby improving corrosion resistance.

(5) $$R_{\mathrm{corr}}=a+b\phi^{-\frac{1}{2}}$$

#### 2.4.2. Dispersion Strengthening

**Figure 6:**
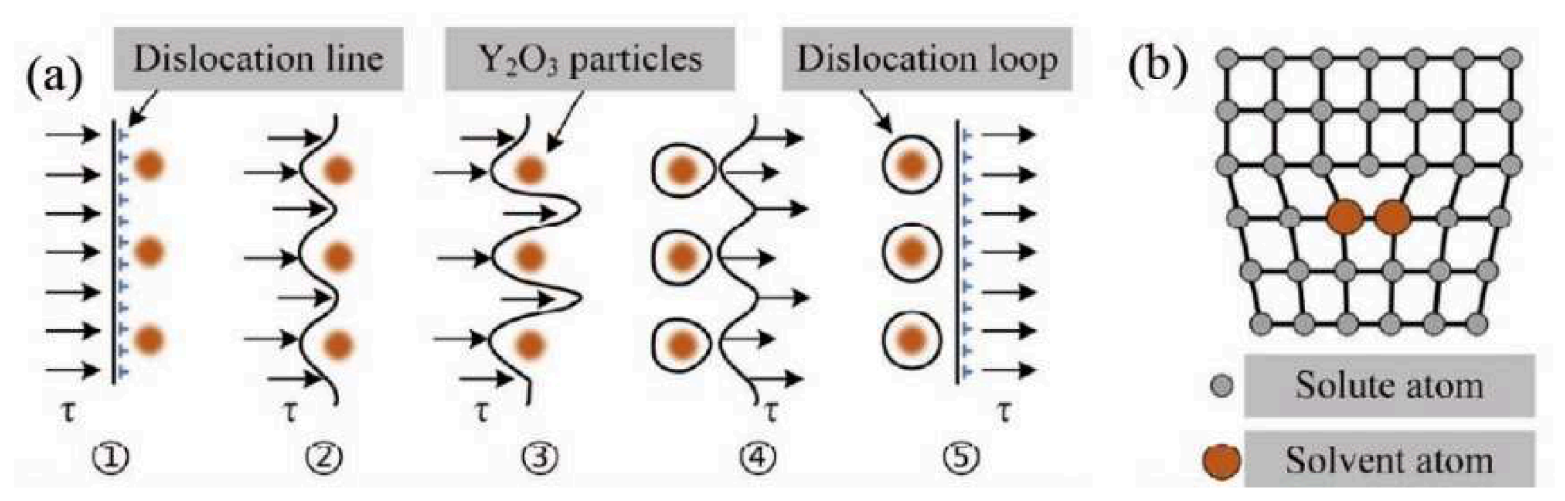
(**a**) Schematic diagram of dislocations bypassing Y_2_O_3_ particles. The direction of the arrow is the direction of the shear stress.; (**b**) schematic diagram of the pinning effect generated by solute atoms [51].

Hard phase particles are typically dispersedly distributed in the matrix in the nano- or submicron scale, exerting a strong pinning effect on the dislocations moving along slip planes. Because these non-shearable particles cannot be cut through, the dislocations must consume more energy to bypass them and leave behind dislocation loops around the particles, producing a dispersion strengthening effect [49,50,51], whereas fine hard phase particles prefer to segregate at grain boundaries, reducing the grain boundary energy and exerting a Zener pinning effect to hinder grain boundary migration (Figure 6). Changjiang Zheng et al. [52] introduced CeO_2_ into the Ni62-Al_2_O_3_-SiC powder system; they found that in addition to enhancing the fluidity of the molten bath, CeO_2_ also facilitates the uniform distribution of hard phases within the coating, produce a dispersion strengthening effect. When crack extension encounters these hard particles, the behavior manifests as penetration, deflection, branching or pinning, which effectively disperses stress concentration and impedes crack growth [53]. The deformation instability zones induced by forming defects are reduced; the mechanical properties of the material are improved [54]. This demonstrates that dispersion strengthening and the pinning effect typically occur simultaneously. Specifically, the dislocations that were unable to cut through are manifested macroscopically as the obstruction of crack propagation, while the pinning effect brings about grain refinement.

In conclusion, rare earth oxides can comprehensively optimize the microstructure from the physical and chemical behaviors of molten bath. Dispersion strengthening and solid solution strengthening can collectively enhance the mechanical properties, wear resistance, and corrosion resistance of the cladded coatings, which demonstrate their remarkable effectiveness on the coating modification.

## 3. Effects of Different Rare Earth Doping on the Forming Quality

**Figure 7:**
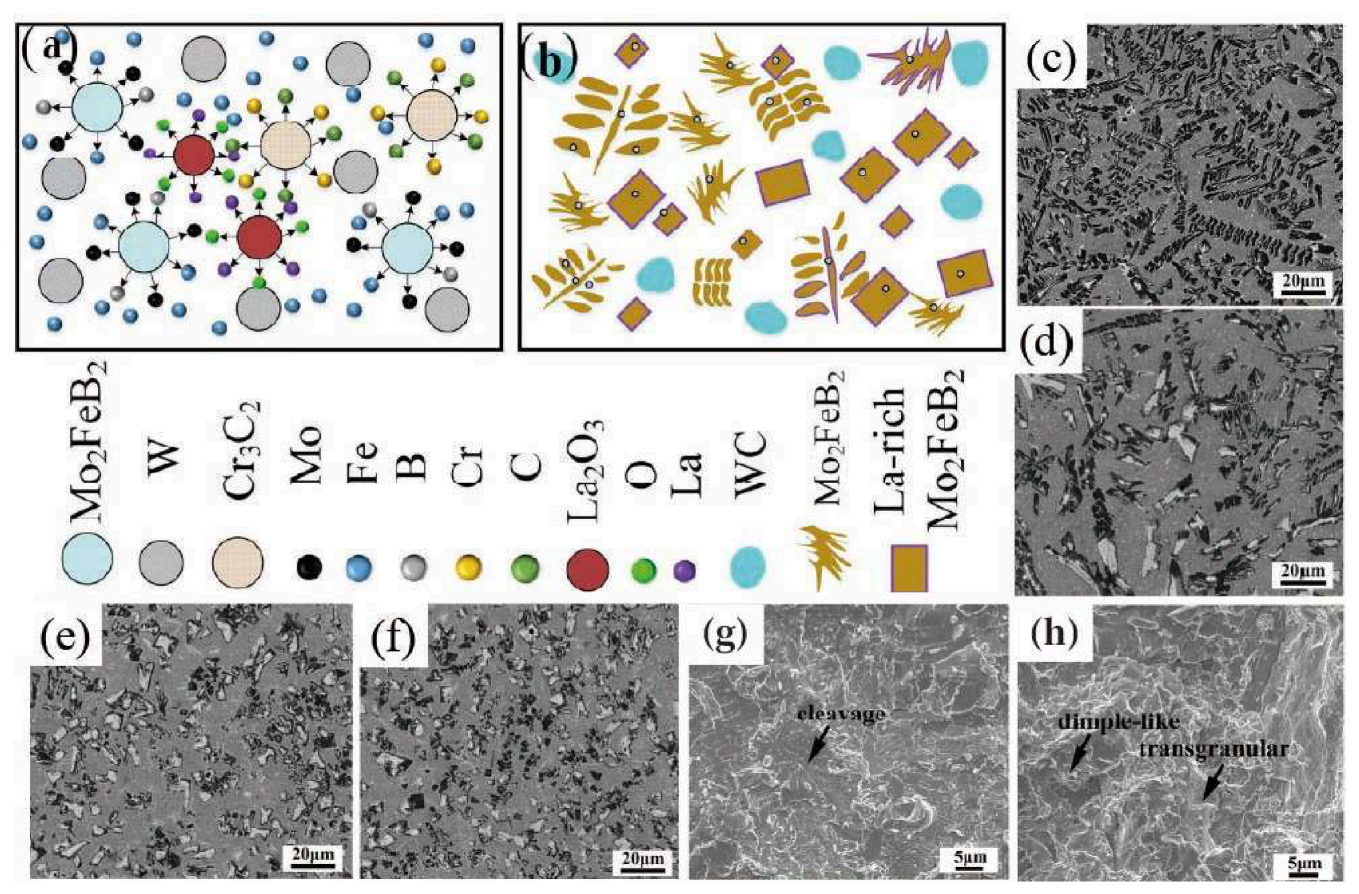
(**a**,**b**) Schematic diagrams illustrating the microstructure evolution of the coating after the addition of La_2_O_3_; microstructure of the middle zone of the coatings with La_2_O_3_ additions of (**c**) 0 wt.%, (**d**) 0.1 wt.%, (**e**) 0.2 wt.%, (**f**) 0.3 wt.%; tensile fracture morphologies of the composite coatings with La_2_O_3_ additions of (**g**) 0 wt.%, (**h**) 0.3 wt.% [55].

In actual service environments, wear and corrosion often occur simultaneously, and the quality of coating formation plays a crucial role in this case. A coating with high formation quality concurrently possesses high density, strong bonding strength, and good uniformity. It not only prevents the penetration of corrosive media but also resists damage from mechanical wear. Consequently, controlling the formation quality of the material is the key point to enhancing the comprehensive performance of the coating. Many coatings exhibit high crack susceptibility and brittleness issues, for example, Hao Zhang [55] et al. discovered that 1.2 wt.% La_2_O_3_ can significantly optimize the performance of Mo_2_FeB_2_-based composite coatings. As illustrated in Figure 7a,b, La atoms are segregated at the grain boundaries of the hard phase particles, exerting pinning and dragging effects on grain boundary migration to hinder their preferred growth. In this way their morphological structure transitioned from the fishbone-shaped to a non-directional, fine and uniformly distributed blocky structure (Figure 7c–f). This increases the grain boundary density and the overall grain boundary energy, requiring crack propagation to absorb more energy to traverse the boundaries. The coating containing 0.3 wt.% La_2_O_3_ exhibited the highest fracture toughness of 14.35 MPa·m^1^/^2^. Fractographic analysis of the tensile fracture surfaces in Figure 7g,h revealed a distinct dimpled morphology in the 0.3 wt.% La_2_O_3_-doped coating, indicative of localized plastic deformation. In contrast, the undoped Mo_2_FeB_2_-WC coating displayed a smooth, brittle cleavage fracture surface. The more uniform and refined microstructure in the La_2_O_3_-doped coating effectively mitigated local stress concentrations and enhanced the grain boundary’s ability to impede dislocation motion, resulting in an increased resistance to elastic deformation at the macroscopic level. Additionally, the incorporation of rare earth oxides facilitated melt purification and promoted superior metallurgical bonding with the matrix. This led to a reduction in impurity segregation, inclusions, and porosity, thereby improving matrix continuity and overall densification. Consequently, the effective load-bearing cross-section per unit volume was enlarged. Furthermore, the enhanced interfacial bonding prevented relative sliding between the coating and the substrate, thereby preserving the structural stiffness of the overall system. Moreover, the anisotropy of material properties is weakened, and the overall forming quality of the coating is improved. The propensity for hard phase particles to peel off under wear is reduced, which established a balance among the material’s hardness, toughness, and wear resistance. But if the strengthening phase is excessive it will reduce the bonding strength and material toughness of the coating, and brittleness will increase [56]. This process reflects the mechanism of rare earth elements achieving defect control through regulating grain structure morphology. The hard phases themselves possess a high elastic modulus. The uniformly distributed, blocky hard phases form a more effective load-bearing skeleton within the coating, which reduces local weak zones caused by uneven distribution or orientation. Consequently, this enhances the overall stiffness and deformation resistance of the coating.

Ni60A-WC coatings have been widely applied because they excellently combined the favorable processability of nickel-based self-fluxing alloys and the ultra-high wear resistance of WC. But their thermal sensitivity and microstructural inhomogeneity limit the upper bound of coating performance [57].

Zhikun Weng et al. [58] in situ introduced various contents of La_2_O_3_ into the Ni-WC coating. According to their research, 0.6 wt.% to 0.8 wt.% La_2_O_3_ can maximize the molten bath fluidity, promote gas and impurity expulsion to reduce internal porosity, and enhance elemental interdiffusion [59]. The microstructural uniformity improves and local overheating is alleviated. Simultaneously, the grain size was significantly refined, leading to an increased number of grain boundaries per unit volume. These boundaries act as effective barriers to dislocation motion, thereby enhancing the yield strength and hardness of the material in accordance with the Hall–Petch relationship. Furthermore, a portion of the undissolved La_2_O_3_ particles reacted within the molten bath, generating fine, uniformly dispersed rare earth compound particles. These hard phase nanoparticles, which cannot be directly penetrated by dislocations, substantially elevated the flow stress required for plastic deformation, consequently increasing the material’s resistance to wear and deformation. Additionally, the material exhibited enhanced stiffness during elastic deformation, making it more difficult to undergo plastic deformation and surface wear. It also improves the wettability between the molten Ni alloy and WC hard phase particles, with the defects such as porosity and lack of fusion reduced. The coating exhibited a more uniform and denser microstructure. This microstructural refinement significantly enhanced both the overall load-bearing capacity and fracture resistance of the coating. It also enables the molten metal to better encapsulate the WC particles, slowing their dissolution rate and reducing stress concentration points, and the surface of the WC particles was covered by a metallurgically sound interfacial layer [60]. These can make sure the efficient load transfer from the matrix to the high-stiffness WC particles, thereby achieving a synergistic load-bearing effect. It also prevents the WC particles from being pulled out or dislodged from the matrix under mechanical loading, which significantly enhances the coating’s resistance to wear and impact. Moreover, this approach slows down the dissolution rate of WC particles and mitigates stress concentration, consequently reducing crack susceptibility. A larger proportion of WC particles are retained as a reinforcing skeleton embedded within the matrix, which suppresses the excessive formation of brittle η phases—such as Co_3_W_3_C and Fe_3_W_3_C—that typically arise from severe WC dissolution. In addition, the preservation of intact WC particles allows the coating to better exploit its inherent wear resistance, while simultaneously avoiding embrittlement. As a result, the coating exhibits improved resistance to crack initiation and propagation.

**Figure 8:**
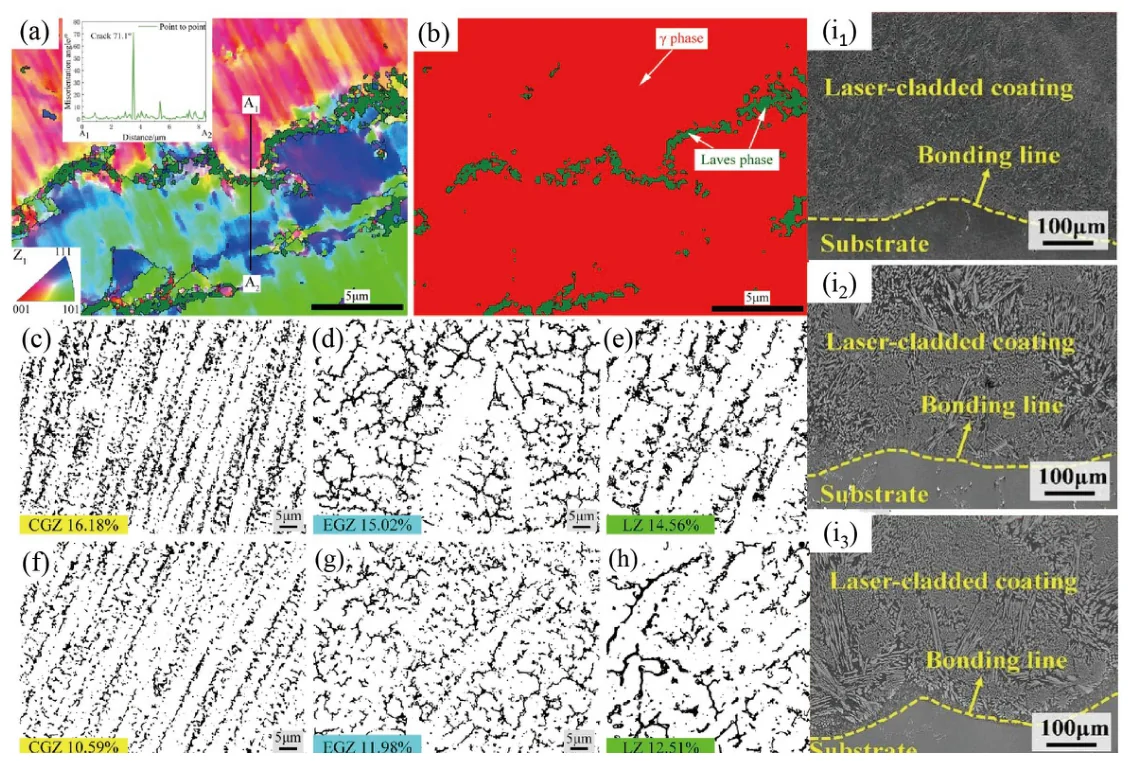
Precipitated phase in the liquation crack of Ni625 cladding layer: (**a**) inverse pole figure and misorientation angle measured along A1–A2; (**b**) phase map. (**c**–**h**) Area fraction of Laves phase in the three representative zones: (**c**–**e**) Ni625; (**f**–**h**) Ni625 + 0.2 wt.% CeO_2_ [32]; (**i1**–**i3**) BSE images at bonding interface with 0 wt.% Y_2_O_3_, 1 wt.% Y_2_O_3_, and 2 wt.% Y_2_O_3_, respectively [61].

During laser cladding of Inconel 625 nickel-based alloy, liquation cracks are prone to occur in the lap zone. Lisheng Zhang [32] et al. added different contents of CeO_2_ to refine the grain size and reduce the precipitation of brittle phases. As shown in Figure 8a–h, before adding CeO_2_, the crack sources were continuously distributed along coarse grain boundaries, consisting of γ + brittle Laves eutectic phases and composed of Mo and Nb elements. After the addition of CeO_2_, the grains were refined, and more alloying elements dissolved into the γ-Ni matrix through solid solution, while the segregation of solute elements such as Mo and Nb was improved. The precipitation of the brittle Laves phase reduced, which enhanced coating hardness and improved wear resistance. The T-800 + Si coating suffers from low-temperature brittleness issues. Guangtai Zhang [61] et al. employed substrate preheating to alleviate thermal stress and introduced Y_2_O_3_ to promote the precipitation of hard Laves phases, effectively compensating for the element dilution resulting from the pretreatment. Additionally, Y_2_O_3_ can react with the Si element in the DD5 coating to form a secondary strengthening phase (Y_5_Si_3_). The stress mismatch between Y_5_Si_3_ and the matrix disperses stress concentration and induces significant dislocation accumulation, thus inhibiting micro-damage accumulation to achieve defect control. In addition, as shown in Figure 8(i1–i3) the interface between the substrate and cladding layer is continuous and smooth, free of cracks and delamination. This illustrates that Y_2_O_3_ facilitates the formation of a gradient transition layer and resolves the high crack susceptibility issue caused by the mismatch in thermophysical properties. These practices can demonstrate that the diverse effects arising from adjustments in the proportion of relevant chemical components generated by rare earth elements can influence the material forming quality.

## 4. Effects of Rare Earth on Coating Properties

### 4.1. Effect on the Microhardness

Mechanical components frequently experience damage modes including wear, contact fatigue, fretting damage, and corrosive wear during service. To improve component service life and adaptability to working conditions, many researchers choose to improve the coating support, delaying surface spalling to strengthen the resistance for initial damage by the means of the enhancement of material hardness [62].

**Figure 9:**
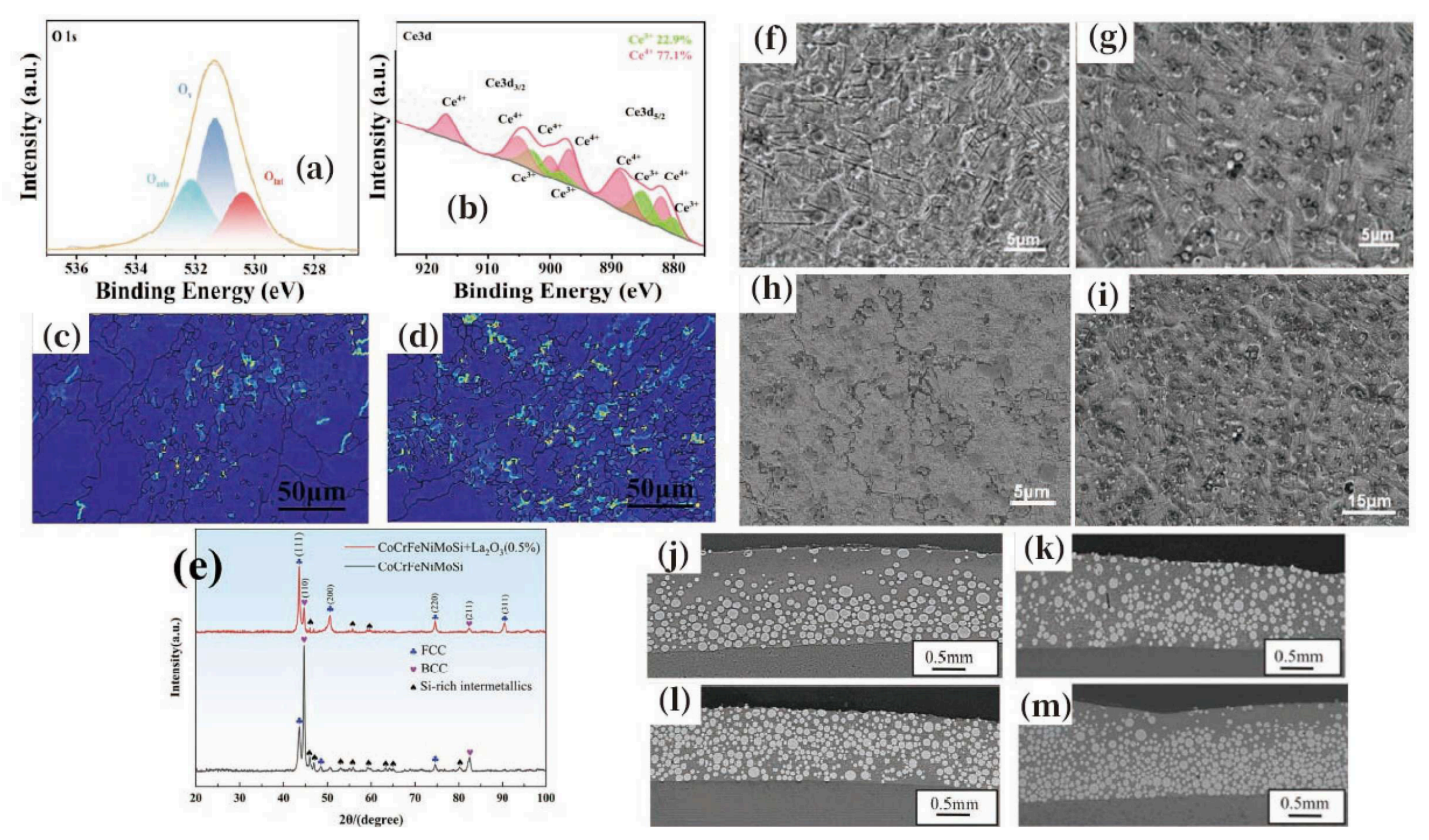
High-resolution XPS spectra of the 2 wt.% CeO_2_ cladding layer: (**a**) O 1 s spectrum, (**b**) Ce 3d spectrum [63]; (**c**,**d**) KAM maps. (**e**) XRD patterns of the coatings [67]. (**f**–**i**) Cross-sectional morphologies: (**g**) 3.0 wt.% Cr + 0 wt.% CeO_2_, (**h**) 3.0 wt.% Cr + 0.25 wt.% CeO_2_, (**i**) 0 wt.% Cr + 0.25 wt.% CeO_2_. (**f**) Secondary electron micrograph of 3.0 wt.% Cr + 0.25 wt.% CeO_2_ [68,72]; (**j**) 0% La_2_O_3_, (**k**) 0.5% La_2_O_3_, (**l**) 1.0% La_2_O_3_, (**m**) 2.0 wt.% La_2_O_3_ [73].

Yiqun Tang et al. [63] prepared Stellite6 alloy composite coatings with different additions of CeO_2_ and detected that the decomposition of CeO_2_ releases oxygen, which signifies the loss of lattice oxygen atoms (O_lat_) and the formation of oxygen vacancies (Oᵥ), which forms a point defect and induces local lattice distortion associated with the Ce^3+^/Ce^4+^ redox cycle at the same time (Figure 9a,b). Furthermore, active Ce^3+^ ions tend to segregate at grain boundaries, then undergo substitutional solid solution, exacerbating lattice distortion due to the size mismatch. Moreover, there is an electrostatic interaction between Ce^3+^ ions and oxygen vacancies, which promotes their spatial correlation. The distortion fields caused by the two are superimposed on each other, forming an elastic stress field that is stronger and more extensive than that of a single defect. As a result, it creates greater resistance to dislocation movement, making it more difficult for the material to undergo plastic deformation and manifesting as a significant increase in the material’s hardness at the macroscopic level (among them, the group with 2 wt.% CeO_2_ has the highest hardness of 587.41, which is about 35% higher than that of the original coating). However, the stress field generated by the solid solution of overabundant rare earth atoms will hinder their own diffusion and result in agglomeration, weakening their grain refinement capability [64,65,66].

Kaitong Guo et al. [67] proposed that in the CoCrFeNiMoSi coating, La can modulate the surface tension and molten bath convection behavior. For this reason, the internal temperature gradient of the molten bath and local solidification rate increases, leading to noticeable grain refinement. This causes an increase in the proportion of high-angle grain boundaries and dislocation density within the coating (as shown in Figure 9c,d), both of which can impede dislocation motion and improve the microhardness [53]. The XRD pattern in Figure 9e clearly shows the existence of the BCC phase and Si intermetallic compounds as the main precipitated phases in the coating. When the material is subjected to an external force, dislocations (line defects in the crystal) move on the slip plane. When they encounter these fine and dispersedly distributed hard precipitated phases, they will act as obstacles to dislocation movement according to the Orowan mechanism, resulting in a dispersion strengthening effect.

These phenomena show that rare earth oxides can impede dislocation motion through solid solution strengthening, while simultaneously enhancing the strength and hardness of the material.

The lattice distortion caused by the substitution of solid solutions of rare earth elements can also serve as the driving force for the eutectic reaction. This promotes the occurrence of phase transformation to improve the microstructure. As demonstrated in the study of Hui Zhang et al. [68], they introduced different contents of CeO_2_ and Cr into Fe-based alloy powders. CeO_2_ causes lattice distortion through the solid solution of Ce atoms, which provides the driving force for the eutectoid reaction of austenite to decompose into α-Fe and Fe_3_C and refine the grains, as shown in Figure 9f,g [69]. The Ce atoms are concurrently dissolute into the α-Fe lattice, which induces the lattice distortion and provides a driving force for the aforementioned process. On the other hand, as shown in Figure 9h,i, the addition of Cr, a ferrite-stabilizing element, lowers the martensitic transformation temperature and accelerates the formation of lath martensite [70,71,72]. The series of changes in the microstructure enable the material to provide stronger support for the hard phase ceramic particles, alleviate stress concentration, and maintain the material’s toughness.

Rare earth elements can promote the homogeneous distribution of hard phase particles within the coating, which is followed with the enhanced structural uniformity and microhardness. Farahmand et al. [73] opted to suppress the dissolution of WC by increasing the scanning speed, while introducing La into the Ni–WC composite to react with elements such as C and O in the substrate, forming new phases of intermetallic compounds and carbides with high hardness. In addition, the high surface activity of rare earth elements facilitates the spreading of Ni atoms on the WC surface and significantly improves the wettability between the liquid Ni and WC particles. Moreover, the temperature coefficient of surface tension for La is considerably higher than that for Ni, which increases the surface tension temperature coefficient of the molten bath and enhances the thermocapillary force, hence promoting mass transfer within the molten bath. Eventually, the carbide particles in the 1 wt.% La_2_O_3_/Ni-WC composite coating fulfill complete uniform and continuous distribution (Figure 9g,h). This ensures the macroscopic consistency of coating hardness; as a result, the soft spots caused by uneven WC distribution are eliminated, and the overall hardness is improved.

**Figure 10:**
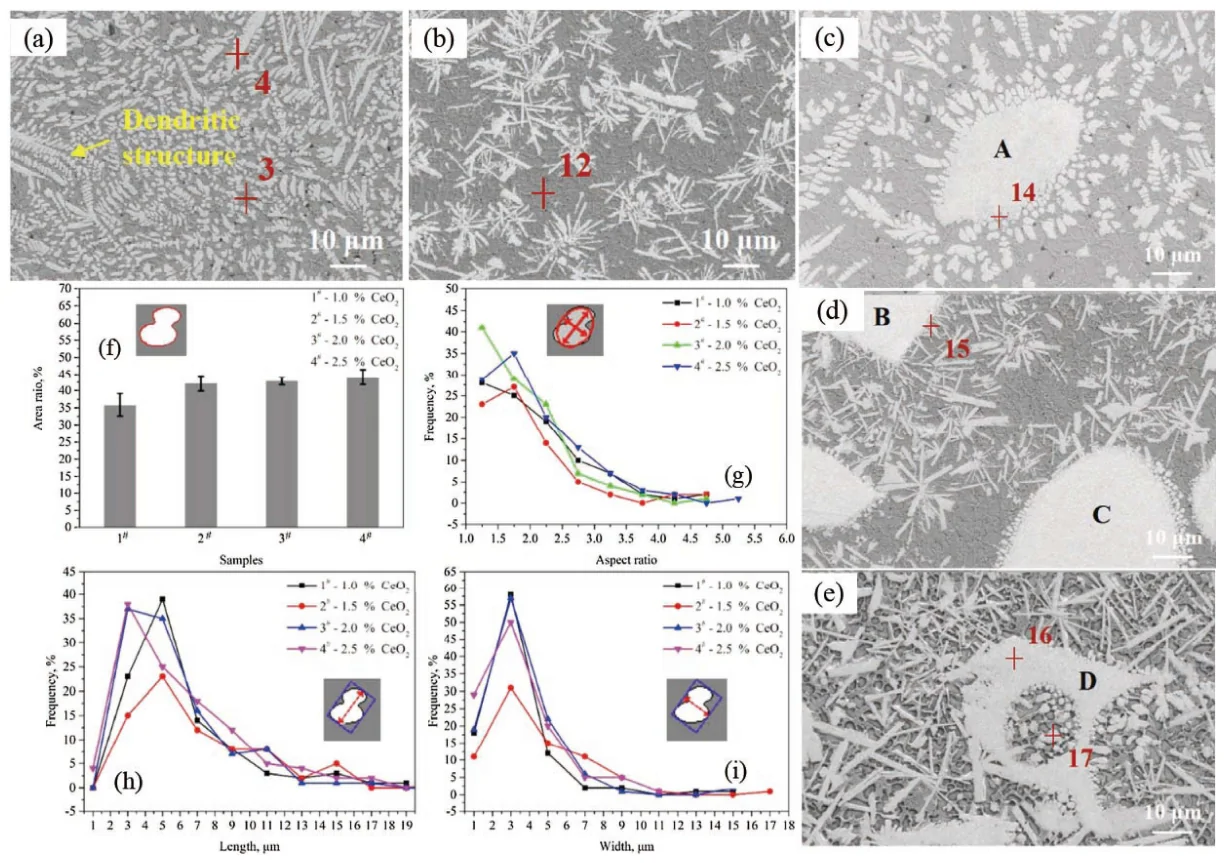
(**a**–**d**) SEM images and WC dissolution behavior of the middle section of Ni60-WC composite coatings before and after adding 1.5 wt.% La_2_O_3_; (**e**) dissolution of WC particles from the middle section of 0.5 wt.% La_2_O_3_ coating [74]; (**f**–**i**) relationship between WC morphology variation and CeO_2_ content: (**f**) area ratio, (**g**) aspect ratio, (**h**) particle length, and (**i**) particle width [60].

The regulating effect of rare earth oxides on the hard phases is also reflected in their influence on the quantity and morphological characteristics. Xiaojin Miao et al. [74] introduced La_2_O_3_ into the Ni60-WC composite coating, which enhanced the thermal permeability of the molten bath and promoted the substrate’s absorption of laser energy. Consequently, the energy acting on WC particles was reduced, and more original WC particles were retained (Figure 10a,b). Concretely, as shown in Figure 10c,d, the softer alloy phases formed by the reverse diffusion of metal atoms into fine WC particles decreased, as did the newly formed fine rod-shaped soft carbides around the original WC particles (Figure 10d, particles around particle B), which used the edges of original WC particles as heterogeneous nucleation cores and displayed a lower hardness. And as illustrated in Figure 10e the dissolution form characterized by internal penetration and outward dissolution was concurrently reduced. Lili Cao et al. [75] and Da Shu et al. [60] are also focused on Ni-WC powder system; they proposed that the composite coating with 2 wt.%CeO_2_ enjoys the best modifying effect. All of this occurs because active Ce atoms are prone to being captured by crystal defects and generate a pinning effect at the grain boundary in order to inhibit the agglomeration and coarsening of WC particles. In the meantime, Ce atoms preferentially capture fine WC particles at the solidification front, enabling their uniform distribution within the cladding layer. Moreover, Ce atoms have a tendency to adsorb onto the WC crystal facets with the highest surface energy, which reduces the surface energy difference among various crystal planes and restricts the rapid preferential growth of high-energy facets [76], achieving the grain refinement and spheroidization in Figure 10f–i. Nevertheless, when the Ce content exceeds 2 wt.%, Ce atoms will form polarization spheres and generate a local stress field that hinders atomic diffusion, finally coarsening the WC particles and thus deteriorating the coating performance. This indicates that CeO_2_ can optimize the morphology of the hard phase, making its distribution more uniform within the matrix and suppressing its dissolution, producing significant enhancement of the material hardness and resistance to plastic deformation.

### 4.2. Effect on the Wear Resistance

When materials are being worn, the cyclic stress will cause the continuous generation of microcracks and eventually result in material spallation. Generally, coating hardness serves as the foundation for wear resistance. Likewise, forming quality is equally critical; it can directly affect the coating’s resistance to cracking and spallation.

**Figure 11:**
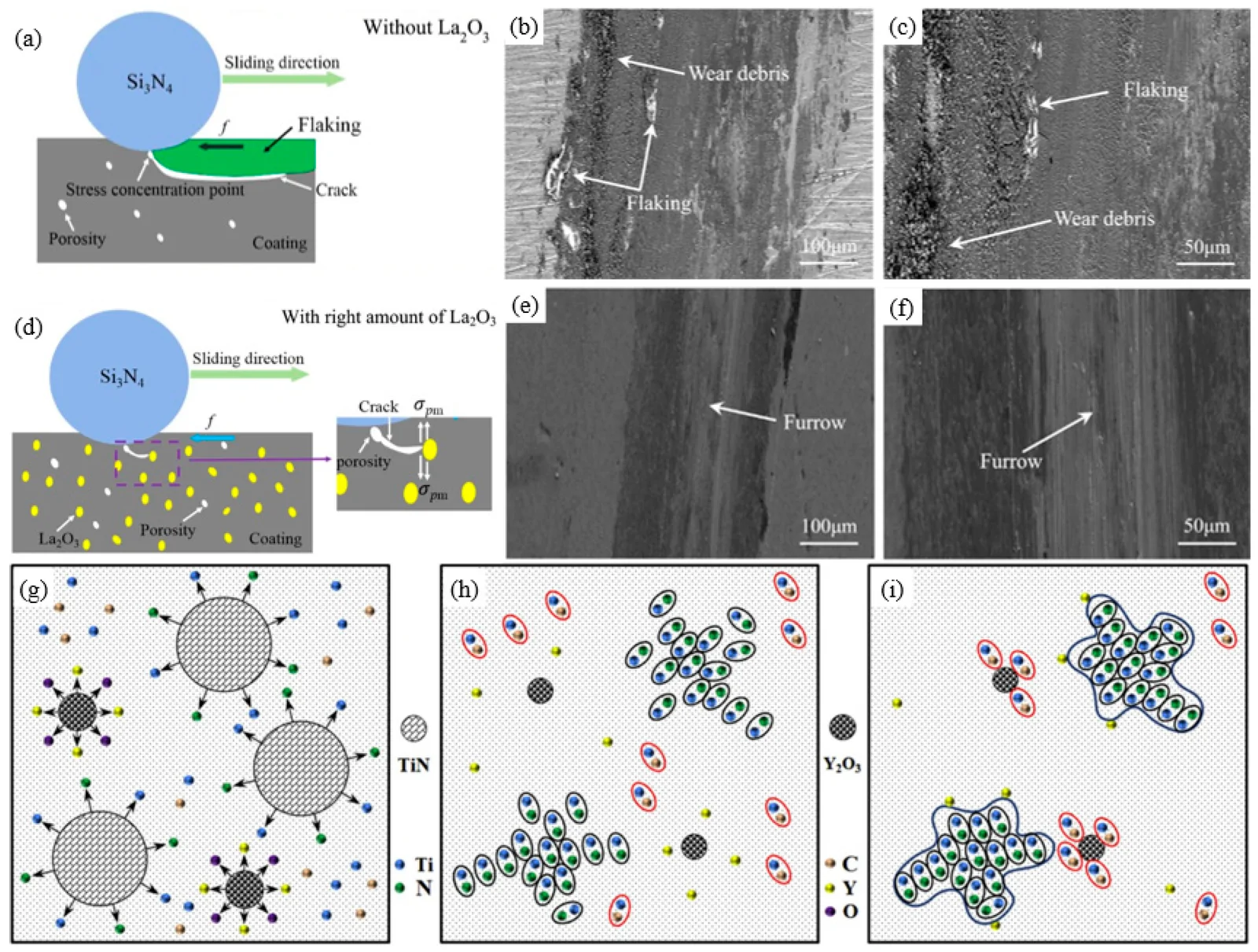
(**a**,**d**) Schematic diagrams of stress analysis of the coating before and after La_2_O_3_ addition, respectively; (**b**,**c**,**e**,**f**) surface wear morphologies before and after La_2_O_3_ addition, respectively [77]; (**g**) dissolution of Y_2_O_3_ and TiN; (**h**) reformation of TiN and in situ formation of TiC; (**i**) growth of TiN and TiC, and distribution of Y atoms [80].

As mentioned in the research of Qiang Wang et al. [77], they prepared 1 wt.% La_2_O_3_/Fe alloy composite coating on the surface of a 45 steel substrate. The initial coating existed some stress concentration points, such as pores (Figure 11a); these stress concentrators evoked microcracks under external loading, leading to severe adhesive wear and abrasive wear, which was accompanied with the surface local spalling (Figure 11b,c). The La_2_O_3_ particles enhanced the material hardness, which follows with the improved plowing resistance that can effectively block the embedment of abrasive ball micro-convex bodies. At the same time, these La_2_O_3_ particles also form a strong metallurgical bond with the matrix. According to the Griffith effect, cracks need to overcome the fracture stress  (σₘ) between the La_2_O_3_ and the matrix to continue the propagation. This makes the coating less susceptible to experience large-area spalling during the friction process, reduces the material removal rate, and shows better wear resistance [78,79] (Figure 11d). As a result, the plowing grooves became shallower, contributed to a smoother worn surface and exhibit mild abrasive wear (Figure 11e,f). This represents that the increase in hardness enhanced the material’s resistance to deformation. Meanwhile, the crack propagation and material spalling are restricted; this leads to a transition in wear behavior and prolongs the duration for which the oxide layer on the coating surface can be maintained.

Fei Weng et al. [80] fabricated a Co42 self-fluxing alloy composite coating with TiN as the reinforcing phase modified by 1.0 wt.% Y_2_O_3_, on the surface of Ti-6Al-4V titanium alloy. As shown in Figure 11d–f, high-hardness phases such as TiC, TiB, and Ti_5_Si_3_ were uniformly distributed within the γ-Co-Ni matrix. Some unmelted Y_2_O_3_ particles acted as heterogeneous nucleation sites during that time, promoting the nucleation of phases such as TiC on their surfaces, and presenting a fixed “core–shell” structure to exert a pinning effect on the surrounding phases, thereby inhibiting their growth (especially TiN dendrites). During the wear process, these refined and pinned reinforcement phases are tightly embedded in the matrix, which can effectively withstand the external load from the counter-grinding wheel and significantly suppress the micro-cutting effect of the abrasive on the matrix.

In addition, the decomposition of Y_2_O_3_ consumed energy, in turn shortening the lifetime of the molten bath, which suppressed the growth of TiN and other phases. This demonstrated that the more uniform and finer microstructure brought about by the rare earth oxide can more effectively resist micro-cutting and spalling during wear, reduce mass loss under dry sliding wear conditions, and enhance wear resistance.

**Figure 12:**
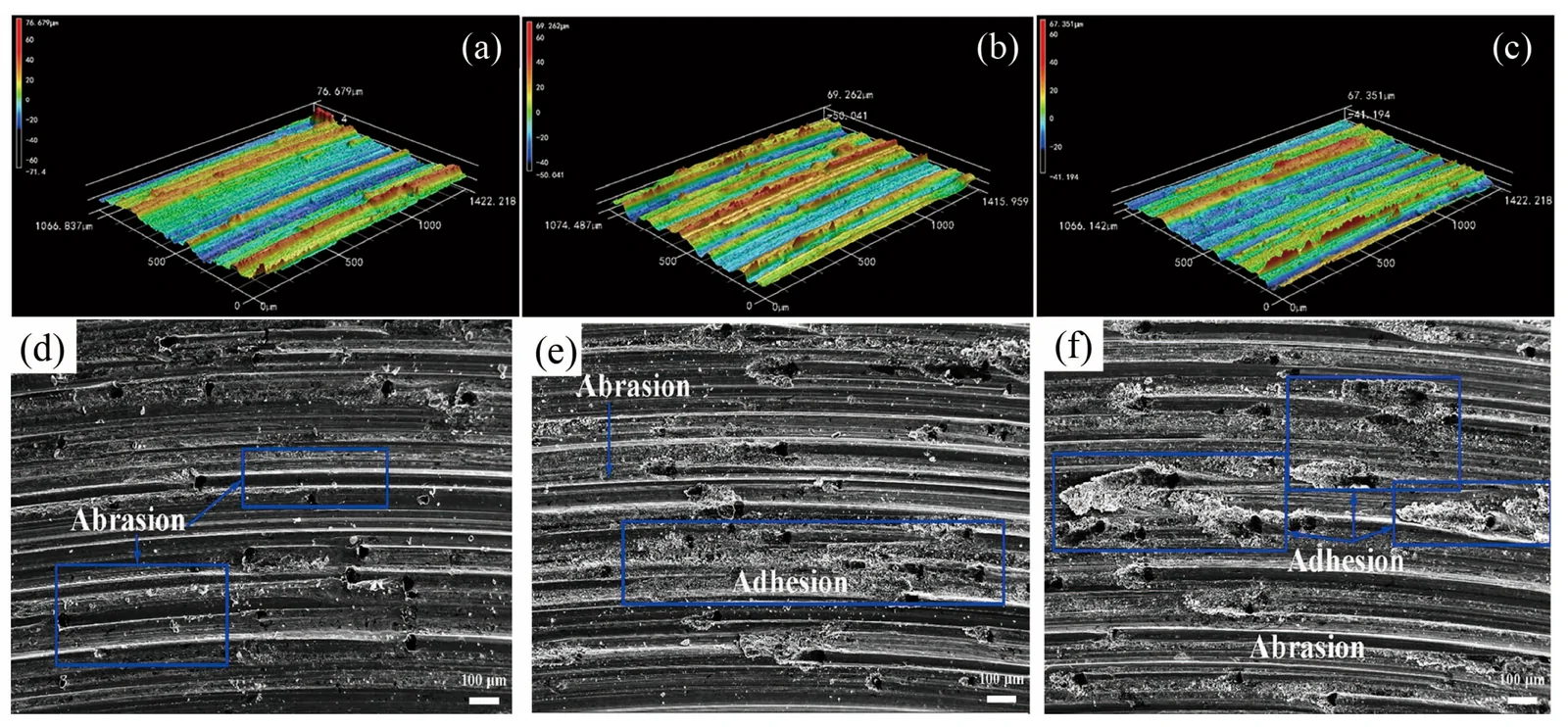
(**a**–**c**) Three-dimensional morphologies of the worn surfaces of pins with different CeO_2_ contents: (**a**) without CeO_2_ addition, (**b**) 0.4 wt.% CeO_2_ addition, (**c**) 0.8 wt.% CeO_2_ addition; (**d**–**f**) corresponding SEM images of the worn surfaces under the respective CeO_2_ contents [81].

However, wear resistance is a complex dynamic property that not only depends on essential factors such as hardness and forming quality, but more importantly on the transition of wear mechanisms. Yanping Wu et al. [81] noticed that CeO_2_ can improve the performance of Fe-based diamond composites by promoting elemental interdiffusion. First, CeO_2_ particles were dispersedly distributed within the liquid Sn phase and enriched at grain boundaries, inducing constitutional undercooling, which facilitated the diffusion of Fe_2_O_3_ from the CeO_2_ surface into the Sn phase. This led to an increase in the content of the intermetallic compound FeSn, bringing a densified internal structure, which improved material’s resistance to plastic deformation [82]. When the CeO_2_ content reached 0.4 wt.%, the coating exhibited the most uniform and dense internal structure and optimal static performance, with a preliminary enhancement in material plasticity. As shown in Figure 12, in this stage, the wear behavior began to transition, and an initial attached layer is formed on the coating surface. The 0.8 wt.% CeO_2_ has facilitated the sufficient interdiffusion of Fe/Sn; for this reason, the plastic deformation capability of the coating improved, and material transfer became more uniform. The wear debris generated under friction was finer; the adhesive wear replaced abrasive wear as the dominant wear mechanism, contributing to superior wear resistance [83]. From these observations, it can be concluded that both the enhancement of material hardness and the improvement of forming quality are ultimately aimed at achieving a transition in wear behavior. Thereupon, optimizing the dynamic wear behavior of a material is often more effective than pursuing higher static mechanical indicators.

Rui He et al. [84] introduced CeO_2_ into the FeCrNiMnAl high-entropy alloy coating. It was found that nano-sized CeO_2_ particles served as heterogeneous nucleation cores, facilitating the transition from columnar grains to fine equiaxed grains, significantly weakening the micro-cutting effect of the friction pair. At the same time, it also promoted the formation and uniform distribution of the BCC solid solution structure, which were followed by the enhancement of the coating microhardness and the reduction in plowing grooves and spalling during the wear process. The addition of CeO_2_ also improved the melt pool fluidity by enhancing Marangoni convection, thereby increasing the internal uniformity and densification of the coating. The changes in the worn surface and abrasive grains are consistent with the phenomena observed by Yanping Wu et al. [81]. Differently, the wear behavior of this coating transitions from severe delamination wear and adhesive wear to milder oxidation wear and abrasive wear. Then, a tribo-oxide layer forms on the worn surface, reducing the wear volume and improving the wear resistance. This phenomenon signifies that, after the transition of wear behavior, the tribo-oxide layer formed on the worn surface can effectively isolate the friction pair from the coating surface, resist the shear force from the friction pair, and thus provide a lubricating and protective function [53,85].

**Figure 13:**
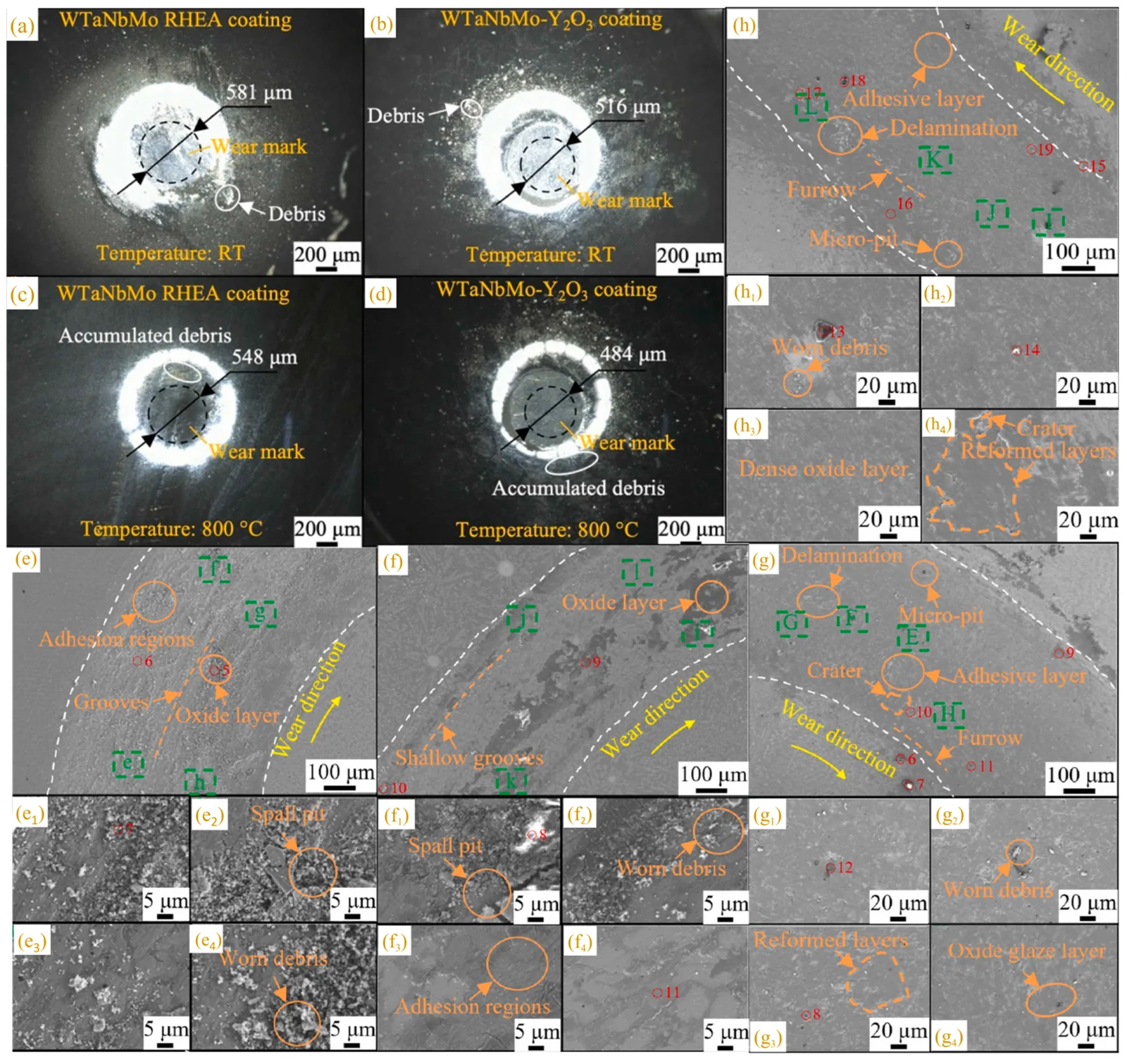
(**a**–**d**) Friction pair images of WTaNbMo coating and WTaNbMo-Y_2_O_3_ coating against the counterpart at room temperature and 800 °C, respectively Morphologies of worn tracks on WTaNbMo-Y_2_O_3_ coatings; (**e**)WTaNbMo RHEA coating at RT, (**e_1_**–**e_4_**) the enlarged images of (**e**); (**f**) WTaNbMo-Y_2_O_3_ coating at RT; (**f_1_**–**f_4_**) the enlarged images of (**f**); (**g**) WTaNbMo RHEA coating at 800 °C, (**g_1_**–**g_4_**) the enlarged images of (**g**); and (**h**) WTaNbMo-Y_2_O_3_ coating at 800 °C, (**h_1_**–**h_4_**) the enlarged images of (**f**) [46].

The influence of rare earth oxides on the formation of the friction oxide layer is also reflected in the experiments of Zhicheng Liu et al. [46]; they developed a WTaNbMo high-entropy alloy coating with different content of Y_2_O_3_, and discovered that Y_2_O_3_ promoted the formation of a fine-grained structure, which firmly bonded to the oxide layer. Simultaneously, the pinning effect of Y_2_O_3_ inhibits grain boundary migration, reduces the diffusion rate of oxygen into the coating interior through grain boundaries, and stabilizes the fine-grained structure as well as the oxide layer thickness [86]. Moreover, the new phases of Y_10_W_2_O_21_ generated from the reaction of Y_2_O_3_ epitaxially grew at grain boundaries, which improved the continuity and interfacial bonding strength between the matrix and the oxide layer, thereby preventing the premature peeling [87]. With the addition of Y_2_O_3_, the material transfer was reduced (Figure 13a–d), which alleviating adhesive wear. Meanwhile, the dense surface oxide layer lowered the friction coefficient and wear rate by means of the self-lubricating effect [88,89] and suppressing adhesive wear and material consumption (Figure 13c,d). However, under room-temperature conditions, insufficient frictional heat generation limited the oxidation reaction, and the lack of lubrication leads to stress concentration, resulting in abrasive wear (Figure 13e and (e_1_–e_4_). But rare earth oxides can promote the formation of the oxide scale and enhance its densification, thereby improving the adhesion of the oxide scale (Figure 13f and (f_1_–f_4_). On the contrary, the wear behavior transitions to oxidative wear under high-temperature conditions because high temperatures promote the occurrence of oxidation reactions (by comparing Figure 13(e_1_–e_4_) with (g_1_–g_4_) and (f_1_–f_4_) with (h_1_–h_4_)), thereby improving plow resistance [90]. At the same time, the cyclic frictional force re-compacts the surface oxide debris back into the worn surface, forming a reformed layer. This study shows that the surface oxide attachment layer on the worn surface can conversely influence the transition of wear mode, hence improving the wear resistance of the material.

### 4.3. Effects on Corrosion Resistance

Corrosion environment is one of the major external factors that conduces to the failure of mechanical components. In a hot corrosion environment, melt deposits can infiltrate the coating, accelerating component fracture [91]. On the other hand, electrochemical corrosion is a localized corrosion caused by electrochemical reactions between the metal surface and ionically conductive electrolyte solution [92]. Prolonged exposure to a corrosive environment will lead to coating failure; hence, researchers are typically improving corrosion resistance to extend the service life of mechanisms.

**Figure 14:**
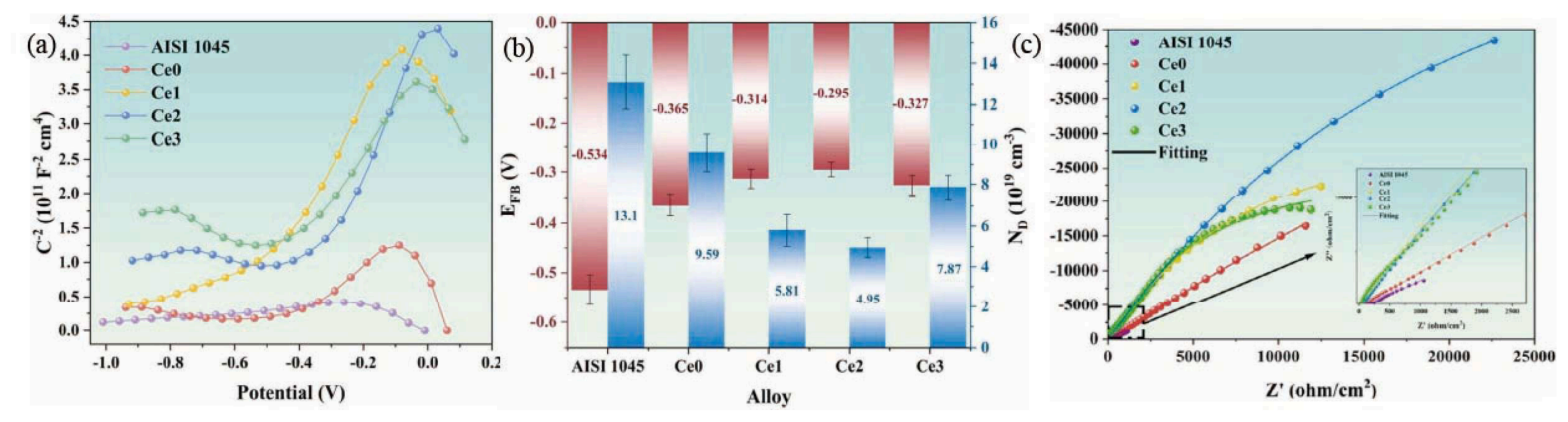
Electrochemical measurements of AISI 1045 and CoCrCu0.5FeNiSi2−CeO_2_ composite coatings in 3.5% NaCl solution: (**a**) Mott-Schottky plot; (**b**) flat band potential EFB and donor density ND; (**c**) Nyquist plot [26].

As WeiBo Gao et al. [26] explained, they prepared CeO_2_-dropped CoCrCu_0.5_FeNiSi_2_ high-entropy alloy coatings on the surface of AISI 1045 steel substrate. Since CeO_2_ can create a thermal barrier effect in the molten bath, it improves the temperature gradient solidification conditions and results in a more uniform and finer microstructure [93]. Consequently, the internal defects caused by elemental segregation are reduced, signifying that the corrosive media penetration channels are also reduced. As shown in Figure 14a,b, the Mott–Schottky curves of all CeO_2_-containing coating samples show positive slopes; their surface passivation film displays n-type semiconductor characteristics. The Ce2 group (containing 2 wt.% CeO_2_) has the lowest ND value, which means that the concentration of free electrons that can participate in the reaction in the passivation film is lower. The height of the bars in Figure 14 represents the absolute value of the hydrogen evolution overpotential for each material. A larger absolute overpotential indicates that the hydrogen evolution reaction is more difficult to occur, implying a lower tendency for hydrogen evolution corrosion when the material acts as a cathode during corrosion. In the figure, the Ce2 coating shows a relatively large absolute overpotential, indicating that the hydrogen evolution reaction is more difficult to proceed, which is beneficial for suppressing the cathodic reaction. The coating enjoys the most compact and uniform structure to effectively inhibit Cl^−^penetration and reduce pitting susceptibility. Although pitting pits were still present on its surface, the reduction in Ce^4+^ to Ce^3+^ endowed the coating with a certain self-healing capability (as shown in Equation (6)). In addition, as observed from Figure 14c, with the CeO_2_ addition, corrosion resistance of the coating initially increases and then decreases when the content exceeds 2 wt.%. This is due to the high chemical activity of Ce, which reduces the Helmholtz free energy required for oxide formation and preferentially diffuses along grain boundaries to the surface to form an oxide layer [94,95,96,97]. In contrast, the superfluous addition gives rise to particle agglomeration, which induces cracks and compromises the integrity of the passive film.

This indicates that rare earth oxides can lower the oxide formation energy, promote the formation of oxide layer, and simultaneously improve the forming quality, thus hindering the diffusion of corrosive media to inhibiting electrochemical corrosion.

(6) $$nCe^{4+}+2M^{n+}+ne^{-}+nH_{2}O\to nCe^{3+}+2nH^{+}+M_{2}O_{n}$$

**Figure 15:**
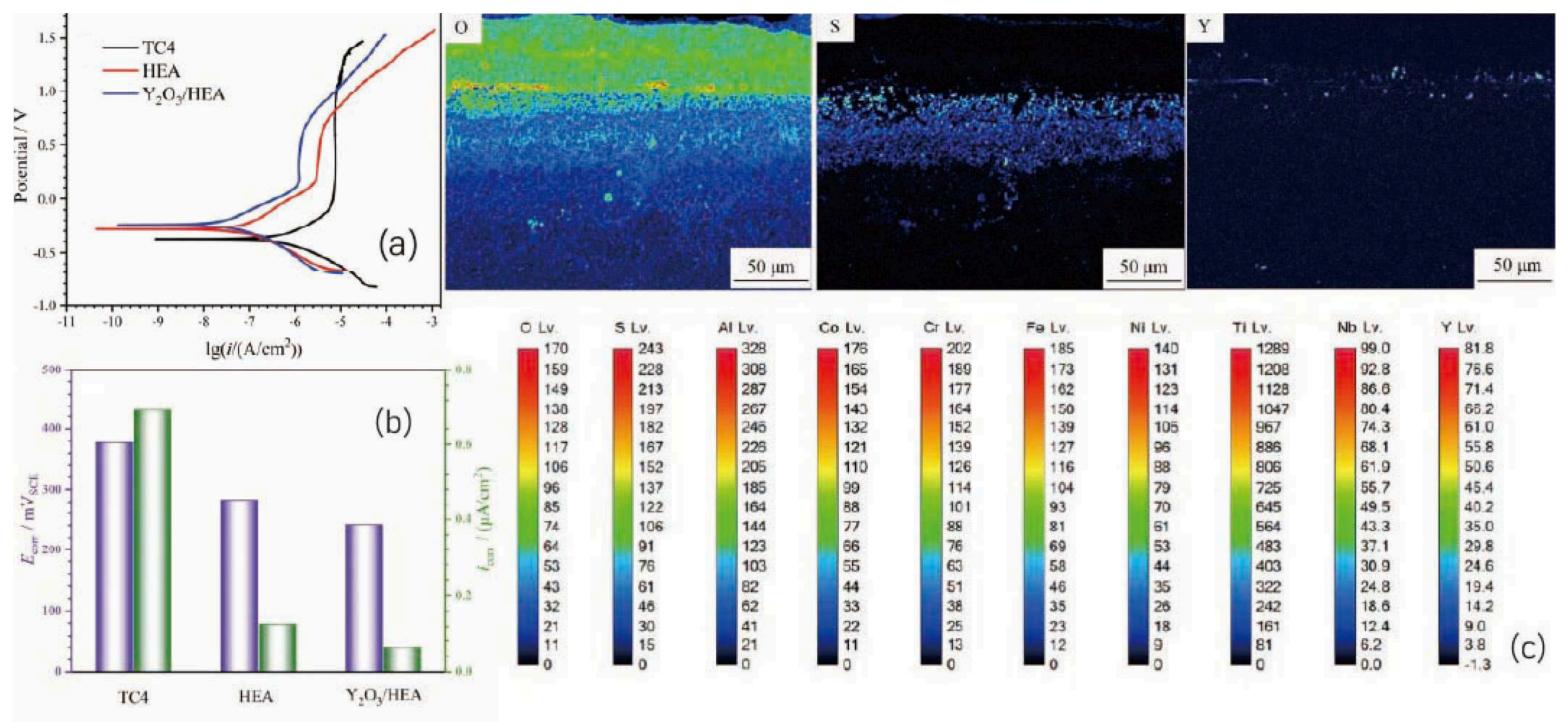
(**a**) Potentiodynamic polarization curves and (**b**) fitting result for the TC4 alloy, HEA and Y_2_O_3_/HEA coatings; (**c**) elemental distribution corresponding to the Y_2_O_3_/HEA-coating microstructure after oxidation at 800 °C for 50 h [98].

The fine and uniform grain structure can also inhibit the occurrence of cathodic reactions in galvanic corrosion. As indicated by Zhen Li et al. [98], they prepared 2 wt.% Y_2_O_3_/CoCrFeNiTiNb composite coating on Ti-6Al-4V(TC4) titanium alloy by laser cladding. Because of the difference in thermal expansion coefficient and interface mismatch between the Y_2_O_3_ particles and the matrix, Y_2_O_3_ introduces heterogeneous dislocation distribution, thereby promoting the nucleation of non-uniform recrystallization and the uniform distribution of dispersed particles. The fine-grained structure provides more high-energy grain boundaries, which offer more active sites for the nucleation and growth of the passive film. The incorporation of Y_2_O_3_ also improves both the compactness and stability of the passive film, thereby more efficiently hindering ionic diffusion and charge transfer. As shown in Figure 15a, the passivation current density (I_g_) decreased to 1.23 µA/cm^2^, merely 40% of that for the HEA coating (3.04 µA/cm^2^). The metal becomes more susceptible to passivation, and the resulting passive film provides stronger protection against corrosion of the underlying metal, favoring the maintenance of the passive state over dissolution in aggressive environments. Simultaneously, it also enhances the substrate’s absorption of laser energy and the interdiffusion of elements, bringing about an increase in the content of TiN ceramic phase and the Cr- and Ti-rich HCP phase. Although the TiN phase always tends to act as a cathodic phase, the refined grain structure weakens the galvanic corrosion and cathodic reaction of galvanic corrosion. In Figure 15b, after adding Y_2_O_3_, the corrosion current density ($i_{corr}$) is the total corrosion rate determined by both the cathodic hydrogen evolution reaction and the anodic metal dissolution reaction. The $i_{corr}$ presents a reduction of approximately 50%. The decrease in the corrosion current density indicates that the reaction rate of the entire electrochemical process is significantly inhibited. The corrosion potential ($E_{corr}$) shifted positively, which indicates that the overpotential of the cathodic reaction increases, and the cathodic reaction is inhibited, thus causing the mixed potential to shift in the positive direction.

The hot corrosion process proceeds via the inward diffusion of O and S elements, forming a distinct diffusion front within the substrate as illustrated in Figure 15c. This occurs because Y atoms segregate at the interface between the oxide layer and corrosion product layer, functioning as effective vacancy traps to suppress the growth of interfacial pores and enhance the density and binding force of the oxide layer, finally reducing the diffusion rate of gaseous oxygen and corrosive media [99,100,101].

In this process, Y_2_O_3_ mitigates galvanic corrosion by refining grain size and promoting elemental interdiffusion. In the meantime, it also enhances the density and uniformity of the oxide layer via defect control, thereby obstructing the penetration and diffusion of corrosive media to retard the electronic and hot corrosion process.

**Figure 16:**
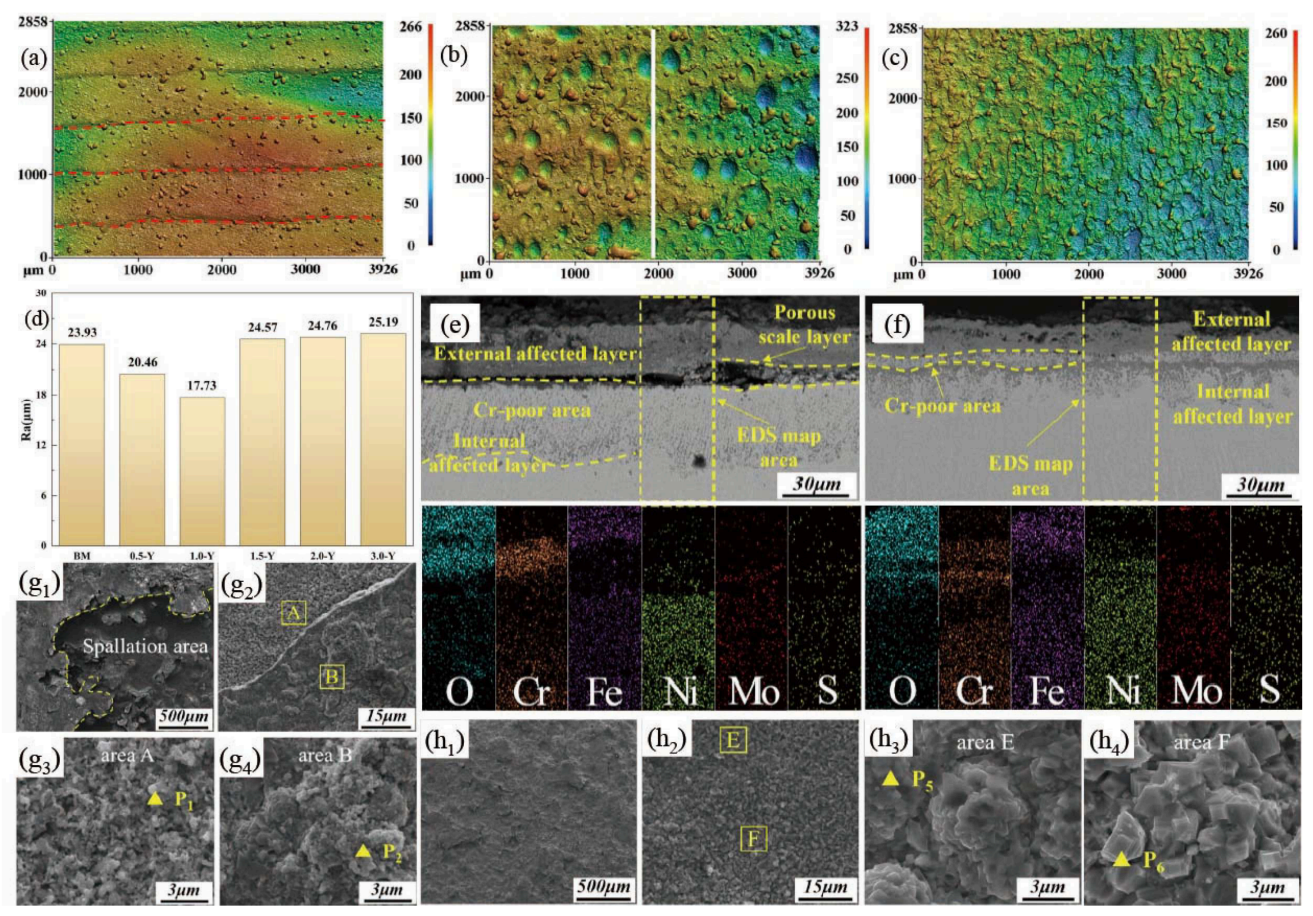
(**a**–**c**) The surface 3D morphology of BM, 0.5−Y, 1.0−Y samples. (**d**) Average roughness values of all samples. (**e**–**h_4_**) Surface micro-morphologies of samples after corrosion in molten salt at 700 °C for 70 h: (**e**,**f**) SEM and EDS analysis results at the interface of BM and 1.0−Y samples, respectively; (**g1**–**g4**) BM sample; (**h1**–**h4**) 1.0−Y sample [102].

Fubing Lin et al. [102] proposed that an appropriate amount of Y_2_O_3_ nanoparticles can reduce melt pool spatter and the formation of defects such as depressions and pores by increasing the viscosity of molten IN718 [103,104,105]. As shown in Figure 16a–c, the IN718 (BM) coating surface has obvious fusion marks (the red dashed line). The addition of Y_2_O_3_ significantly improves the wettability of the molten bath, and strengthens the bonding between the powder and the substrate [106], leading to the elimination of unmelted zones and decreased surface roughness Ra (Figure 16d). Accordingly, the electron work function increases and suppresses the onset of the corrosion process. At the same time, Y_2_O_3_ generates a pinning effect at grain boundaries to effect reverse grain growth and promotes the outward diffusion of Cr to form a dense oxide film, while a Cr-depleted zone appears within the coating. The BM coating displays extensive surface spallation, with cluster-distributed, loose, blocky corrosion products, indicating decomposition of the surface protective oxide layer. After the addition of Y_2_O_3_, there is no significant spallation observed on the surface, and the area of Cr-depleted zone diminishes, which mitigates the corrosion severity. As shown in Figure 16(e–h4), all hot corrosion cross-sections present a distinct layered structure: the surface oxide layer constitute the outer affected zone; the middle region is a porous scale oxide layer. Additionally, the inner region is the substrate corrosion zone. These results suggest that improved surface smoothness can establish a rising energy barrier for corrosion initiation, while the refined grain structure facilitates the diffusion of film-forming elements to promote the formation of an oxide film.

## 5. Effect of Rare Earth Additive Property Parameters on Modification Outcomes

Guangtai Zhang [61] et al. introduced Y_2_O_3_ at concentrations of 0 wt.%, 1 wt.%, and 2 wt.% into the T-800 + Si-DD5 coating. Among these, the coating containing 1 wt.% Y_2_O_3_ exhibited the highest average microhardness and the best wear resistance. This improvement can be attributed to several mechanisms. Yttrium atoms tended to segregate at the dendrite front, which reduced interfacial tension and induced a solute drag effect, thereby inhibiting grain growth and resulting in a fine-grain strengthening effect. Meanwhile, the addition of Y_2_O_3_ promoted the precipitation of fine secondary-phase particles, specifically the Co_3_Mo_2_Si Laves phase, which impeded dislocation motion and contributed to precipitation strengthening. These combined effects enhanced the coating’s resistance to plastic deformation and cutting. Additionally, yttrium was prone to segregation at the interface between the oxide scale and the substrate. There, it combined with impurities to form stable sulfides, preventing sulfur from weakening the interfacial bonding and thereby improving the adhesion of the oxide scale. Due to its high surface activity, yttrium also accelerated the diffusion of molten metal and the elements involved in oxide scale formation. This facilitated the rapid formation of a complete, dense, and continuous oxide layer with strong adhesion. The high strength and hardness of the material further provided better mechanical support for the oxide scale, allowing it to release stress more readily through plastic deformation and reducing the risk of surface wrinkling and spallation. As a result, the wear mechanism shifted from abrasive wear to oxidative wear, significantly enhancing the coating’s wear resistance. However, in the coating with 2 wt.% Y_2_O_3_, excessive and inhomogeneous segregation of yttrium weakened the solute drag effect and led to an excessively high content of the Laves phase. This triggered a rare earth polarization effect and Ostwald ripening, where small grains were consumed by larger ones, resulting in grain coarsening [60,107]. Furthermore, excessive dislocation accumulation and severe lattice distortion or mismatch caused the coating to soften. This destabilized the surface microstructure, making it difficult for the oxide layer to form a continuous coverage [85]. Additionally, excess Y elements preferentially react with film-forming elements such as Si, Mo, and Co, inhibiting local oxidation reactions [108]. Consequently, the quality of the oxide layer deteriorated, leading to a decline in wear resistance.

YuJiang Xie [53] et al. fabricated AlCoCrFeNi_2_._1_ + xCeO_2_ high-entropy alloy coatings, among which the coating containing 1 wt.% CeO_2_ exhibited optimal overall performance. An insufficient CeO_2_ addition yielded limited modification effects, whereas an appropriate amount of CeO_2_ improved melt pool fluidity and promoted convective flow, thereby enhancing elemental homogeneity and reducing segregation. This, in turn, mitigated the susceptibility to intergranular and galvanic corrosion [59]. Concurrently, Ce atoms served as heterogeneous nucleation sites, lowering the nucleation energy barrier. Their segregation at grain boundaries generated a pinning effect that restricted grain growth, leading to significant grain refinement. Consequently, the density of grain boundaries and the proportion of high-angle grain boundaries increased, along with a notable rise in dislocation density, which intensified dislocation interactions [109]. The combined contributions of grain refinement strengthening and dislocation strengthening improved the microhardness of the material, as well as its resistance to plastic deformation and spalling. As a result, the dominant wear mechanism transitioned from fatigue wear to adhesive wear accompanied by minor abrasive wear, yielding a more stable friction coefficient and enhanced wear resistance. Conversely, excessive CeO_2_ addition led to grain coarsening and severe Ce segregation at grain boundaries, disrupting the uniformity of elemental distribution. The formation of elemental agglomerates increased the risk of corrosion, while the resultant inhomogeneous passive film became more susceptible to breakdown, substantially deteriorating corrosion resistance. The non-uniform elemental distribution also reduced dislocation density, thereby compromising both hardness and wear resistance.

The particle size effect of the REE additive is a critical experimental variable. According to the study by Shudan Li et al. [110], composite coatings containing 1 wt.% CeO_2_ with particle sizes of 50 nm, 0.5 μm, and 5 μm were prepared, and significant differences in their effects on coating performance were observed. In the coating with nano-sized CeO_2_, the CeO_2_ particles tended to agglomerate at grain boundaries, resulting in compositional segregation and inhibiting the solid solution process among the constituent elements. Additionally, an α-Ti inclusion phase was prone to form, leading to a poor microstructure and consequently compromising the mechanical properties. In the coating with micron-sized CeO_2_, a large number of unmelted CeO_2_ particles were concentrated at the grain boundaries, which readily induced stress concentration and promoted the initiation of internal cracks. However, due to the relatively weak Coulombic and van der Waals forces between submicron particles, agglomeration was difficult to occur. The submicron CeO_2_ particles were uniformly distributed at the grain boundaries, which significantly refined the grain size, inhibited the precipitation of intermetallic compounds, and promoted the uniform distribution of components as well as the solid solution process, thereby enhancing the structural homogeneity of the coating. Moreover, owing to the significant difference in the coefficient of thermal expansion between the submicron CeO_2_ particles and the high-entropy alloy matrix, a crack bridging effect was generated. This effect consumed and absorbed part of the energy during crack propagation, causing crack deflection or even closure, and most effectively suppressed fatigue crack growth, resulting in an increase in fracture toughness to 148.3 MPa·m^1^/^2^. Furthermore, the CeO_2_ particles themselves are relatively soft and can serve as a lubricant during wear, reducing the micro-cutting effect on the coating. CeO_2_ also enhances the cohesive strength of the oxide film, making it more resistant to rupture and spallation during friction. Among the three particle sizes, the submicron CeO_2_ particles, due to their most uniform distribution and optimal grain refinement effect, produced the most intact oxide film, generated the least amount of wear debris, and exhibited the strongest inhibition of both oxidative wear and abrasive wear.

## 6. Industrial Application Statues and Challenges

Rare earth oxides such as CeO_2_, La_2_O_3_, and Y_2_O_3_ are widely used in practical process applications due to their superior physical and chemical properties.

CeO_2_ possesses a unique reversible valence state conversion capability between Ce^3+^ and Ce^+4^, enabling its surface oxide passivation layer to finish a dynamic self-repair. This property confers exceptional resistance to the oxidative and corrosive environments at elevated temperatures. This enables it to exhibit exceptional resistance to high-temperature oxidation and hot corrosion, meeting the core requirements of extreme operating conditions such as aerospace engine blades and gas turbine hot-section components.

La, as a light rare earth element within the same group, exhibits higher surface activity and a greater atomic radius mismatch, which can produce a more pronounced grain refinement effect under the rapid cooling conditions of laser cladding. Furthermore, La_2_O_3_ possesses a hexagonal layered crystal structure, enabling it to maintain stable chemical properties at elevated temperatures and exhibit excellent solid lubrication functionality. This effectively reduces the friction coefficient and enhances wear resistance, while also contributing to grain refinement and microstructure optimization. Given that components such as mining machinery and molds are primarily subject to wear, impact, and fatigue failure, the outstanding performance of La_2_O_3_ in reducing friction coefficient and improving wear resistance makes it an ideal additive for high-wear working conditions.

Y_2_O_3_ serves as a classic stabilizer for ceramic materials such as zirconia. It promotes the formation of the tetragonal phase in zirconia and utilizes the phase transformation toughening mechanism where the fracture toughness of ceramics is improved by 3–5 times. This characteristic makes it highly effective at suppressing crack propagation in ceramic–metal composite coatings. Furthermore, leveraging the high surface activity of rare earth elements, Y_2_O_3_ helps stabilize the ceramic phase, improves interfacial bonding, reduces the porosity of ceramic coatings, and enhances thermal shock resistance. As a result, it mitigates interfacial cracking and coating delamination caused by mismatches in thermal expansion coefficients between the ceramic phase and the metal substrate, significantly extending the service life of coatings under rapid thermal cycling conditions.

However, this technology still faces some challenges in the engineering application process.

(1) Rare earth compounds may encounter deactivation, migration, or precipitation under service conditions including high-temperature water vapor, thermal shock, and corrosive media, leading to performance degradation [111]. For example, when Y_2_O_3_ is exposed to a high-temperature Na_2_SO_4_-V_2_O_5_ corrosive environment, it will react with VO_3_^−^ to form YVO_4_, which reduces the concentration of the corrosive medium but deactivates the Y_2_O_3_ additive [112,113]. Furthermore, RE ions can also dissolve from the crystal lattice and migrate into the molten salt formed by the reaction of Na_2_SO_4_ and V_2_O_5_ on the coating surface. Subsequently, they form stable new compounds that precipitate out, ultimately conducing to the decomposition of the coating surface [114,115,116].

(2) Under high-temperature environments, rare earth elements may react with the matrix material or environmental contaminants, resulting in phase transformation of material composition and degradation of performance [117]. Accordingly, it is necessary to fully consider the potential adverse reactions when designing powder materials for high-temperature applications. Moreover, certain rare earth compounds exhibit incompatibility with the thermally grown oxide (TGO). For instance, La_2_Zr_2_O_7_ is subjected to interfacial reactions with α-Al_2_O_3_ at temperatures above 1100 °C, resulting in the accumulation of cyclic stress at the interface in the meantime because of the mechanical incompatibility; for all these reasons, it affected the high-temperature service life of thermal barrier coatings [118,119].

(3) When using rare earth oxides for modification, there exists a distinct threshold effect, requiring strict control over the doping amount of the element. Excessive addition not only diminishes the effectiveness of grain refinement and adversely affects the performance of the coating, but also directly drives up production costs. The optimal addition amount can only be determined through testing under different powder systems and process parameters. This has severely restricted the standardization of large-scale universal application of rare earth modification.

(4) Nano-sized rare earth oxide particles are extremely prone to agglomeration, which not only affects the interfacial bonding within the coating but also reduces functional consistency. In addition, in order to ensure production efficiency, the laboratory-scale uniform doping is crucial, but it is difficult to achieve in production lines. This issue becomes even more severe, especially when the addition amount of rare earth elements is relatively high. However, the uneven distribution of rare earth elements often leads to a series of microstructural defects. Therefore, optimization of powder particle size and development of powder mixing processes and laser parameters suitable for practical industrial production remain a technical challenge that needs to be overcome.

(5) Cost and environmental friendliness are also factors that limit the widespread application of rare earth additives: high-purity rare earth materials are expensive, resulting in the cost of rare earth modification to far exceed that of traditional coating materials. Therefore, for rare earth elements with high oxidizability and susceptibility to oxidation, the relevant process parameters need to be optimized. Specifically, the melting speed and laser power should be appropriately reduced to avoid high-temperature volatilization and oxidation failure, thereby reducing material loss [120]. In addition, rare earth minerals such as monazite and xenotime contain radioactive elements; the processing wastewater may also produce high concentrations of heavy metal ions [121,122,123]. Accordingly, a complete rare earth element recovery system needs to be established to prevent environmental pollution and reduce related production costs. This can not only prevent the environment from being contaminated, but also lower the associated production costs [124].

To sum up, the current approach of rare earth oxide modification is beset by numerous limitations in large-scale industrial applications. It is urgently necessary to develop cost-effective composite powder systems and design relevant standardized parameter settings, and consequently further expand the application scope and efficiency of rare earth additives in industrial production.

## 7. Conclusions and Prospect

This article summarizes the recent experimental research progress on the modification of different rare earth (RE) additives coatings via laser cladding. It also elaborates on the practical applications and action mechanisms of rare earth elements in the laser cladding technology from several aspects, like coating-forming quality, microhardness, wear resistance and corrosion resistance, and discusses their intrinsic connections. The main conclusions of this article are as follows:

(1) The basis of RE oxides improving coating performance is grain refinement driven by their high surface activity. First, they reduce the nucleation work by lowering the surface tension of the molten metal and act as heterogeneous nucleation substrates to increase the nucleation rate, promoting the transition from columnar grains to equiaxed grains. Second, RE atoms exert pinning and dragging effects on grain boundaries, concurrently improving molten bath convection and increasing the local solidification rate to inhibit grain growth. Quantitative analyses indicate that optimal RE additions typically range from 0.5 to 2.0 wt.%, beyond which particle agglomeration compromises the refinement effect and may even degrade coating properties.

(2) Rare earth elements can also control interface-forming defects by promoting the precipitation of strengthening phases and reacting with inclusions. Meanwhile, they reduce surface tension to regulate molten bath behavior, mitigate elemental segregation, decrease crack sensitivity and improve coating uniformity and smoothness. Thus, the fracture toughness and frictional resistance of the coating are improved. In addition, rare earth atoms segregate at interfaces and form chemical bonds, bringing a stronger metallurgical bonding and enhanced interfacial continuity. Moreover, rare earth elements can hinder the preferred growth of grains through pinning and dragging effects, weakening structural anisotropy and improving the corrosion resistance of the material for the overall forming quality of the coating.

(3) Introducing rare earth additives in the coating will bring the solid solution strengthening effect and fine-grained structure, directly increasing the coating hardness (typically by approximately 10–30% compared to unmodified coatings). Rare earth elements also promote phase transformation processes, thus improving the microstructural morphology and providing better support for hard phases. In addition, rare earth atoms adsorb onto the surfaces of hard phases, hindering their preferred grain growth and optimizing the morphology. Simultaneously, they improve the wettability between different components, promote the internal substances transportation, and slow down the dissolution rate of hard phases. As a result, the stability and uniformity of the hard phases are enhanced, and the material hardness is improved.

(4) The increase in material hardness induced by rare earth elements will in turn enhance the plow resistance, enabling the coating to strongly impede the embedment of abrasive balls; the surface plowing grooves and spalling are then reduced. A more compact and uniform coating structure also improves the material’s plastic deformation capacity, which provides better mechanical support for the formation of the surface oxide film and enhances the adhesion of the surface oxide film. These cause the abrasion material transfer to be more uniform and the wear debris to be finer. Additionally, the enhanced interfacial bonding allows for more effective resistance against cutting and spalling during the wear process. These mechanisms collectively promote a transition in the coating’s wear behavior and improve wear resistance.

(5) The addition of REO improves the forming quality of the material, resulting in a finer and more uniform microstructure. This reduces the tendency toward galvanic corrosion and intergranular corrosion while promoting internal elemental diffusion, lowering the energy required for oxide formation. Furthermore, REO can react with impurities to enhance the adhesion of the oxide film, making it less prone to spallation. This promotes the formation of a complete and dense surface oxide film, thereby reducing the diffusion rate of corrosive media such as oxygen. In addition, REO can reduce surface roughness, eliminate incomplete fusion zones on the surface, and improve the initiation conditions for corrosion reactions, thereby enhancing the corrosion resistance of the material and extending the service life of components in corrosive environments.

(6) RE elements play a momentous role in modifying the performance of laser-cladded coatings. Currently, the REO additives commonly used for laser-cladded coatings are La-, Ce-, and Y-based compounds, while addition of other rare earth compounds and pure rare earth elements is infrequently applied. Future research opportunities include (i) systematic investigation of RE elements beyond La, Ce, and Y; (ii) expanded research on other less-studied coating systems to fill the current application gap; (iii) comprehensive evaluation of mechanical properties beyond hardness, including fracture toughness, fatigue resistance, and functional properties such as magnetic performance; (iv) development of cost-effective composite RE powder systems and standardized process parameter settings; (v) in situ monitoring and computational modeling of RE-modified molten bath dynamics to achieve predictive process control; and (vi) investigation of RE additive deactivation mechanisms and mitigation strategies under extreme service conditions.

## Figures and Tables

**Table 1:** The average grain sizes and grain size distributions of the coatings with different contents of LaB6 addition.

LaB_6_ Content	0.0 wt.%	1.5 wt.%	3.0 wt.%	4.5 wt.%
Average grain sizes	2.04 µm	1.44 µm	0.99 µm	1.13 µm
Grain size distributions	51.18%	75.02%	88.55%	79.36%

## Data Availability

No new data were created or analyzed in this study. Data sharing is not applicable to this article.

## References

[1] La Monaca A., Murray J.W., Liao Z., Speidel A., Robles-Linares J.A., Axinte D.A., Hardy M.C., Clare A.T. (2021). Surface Integrity in Metal Machining—Part II: Functional Performance. Int. J. Mach. Tools Manuf..

[2] Bartošák M., Horváth J. (2024). A Continuum Damage Coupled Unified Viscoplastic Model for Simulating the Mechanical Behaviour of a Ductile Cast Iron under Isothermal Low-Cycle Fatigue, Fatigue-Creep and Creep Loading. Int. J. Plast..

[3] Zhang W., Chen A., Yu Y., Li J. (2025). Investigation into the Erosion Damage Mechanism of High-Temperature, High-Pressure, and High-Speed Gasflow on Metal Surfaces. Eng. Fail. Anal..

[4] Tian M., Choundraj J.D., Voisin T., Wang Y.M., Kacher J. (2022). Discovering the Nanoscale Origins of Localized Corrosion in Additive Manufactured Stainless Steel 316L by Liquid Cell Transmission Electron Microscopy. Corros. Sci..

[5] Li J., Liu Z., Ma H., Liu Q., Mao J., Zhang J., Kong Y. (2022). Comparative Study on the Corrosion Behaviour of 1.4529 Super Austenitic Stainless Steel and Laser-Cladding 1.4529 Coating in Simulated Desulfurized Flue Gas Condensates. Corros. Sci..

[6] Hidalgo Viteri J., Cotolan N., Lupan A., Brânzanic A.M.V., Turdean G.L. (2024). A Poly(Methyl Methacrylate)–Ibuprofen Composite Film as Anticorrosive Coating of Ti–6Al–4 V Surface. J. Solid State Electrochem..

[7] Li Y., Džugan J. (2025). Interface Structures and Load-Bearing Mechanical Properties in Additively Manufactured Metallic Functionally Graded Materials. Mater. Sci. Eng. A.

[8] Huang Y., Chen X., Wu W., Wang Y., Wang Y. (2025). Effect of Laser Remelting on the Microstructure and Properties of an AlCoFeNi Eutectic High-Entropy Alloy Fabricated through Double-Wire Arc Additive Manufacturing. J. Alloys Compd..

[9] Li J., Guo Q., Tang Q., Zhao G., Li H., Ma L. (2024). Effect of Electron Beam Remelting on Microstructure and Wear Properties of HVOF Ni/WC Coatings. Wear.

[10] Manjhi S.K., Bontha S., Balan A.S.S. (2024). Enhancing Fatigue Performance of AZ31 Magnesium Alloy Components Fabricated by Cold Metal Transfer-Based Wire Arc Directed Energy Deposition through LPB. J. Magnes. Alloys.

[11] Zhang X., Huang S., Li D., Geng J., Yang F., Li Q. (2022). An Approach to Improve the Microstructure and Mechanical Properties: A Hybrid Manufacturing of Laser Directed Energy Deposition and Shot Peening. Addit. Manuf..

[12] Bag P.P., Roymahapatra G. (2021). Surface Engineering for Coating. Advanced Surface Coating Techniques for Modern Industrial Applications.

[13] Tang J., Li Z.-S., Liu X.-B., Chen G.-D., Liu F., Wang S., Meng F.-G., Wang D.-S., Wang K.-M. (2026). Tribology and Oxidation Properties of Graphite/CuNiTiCrNb HEA Coatings Produced by Laser Cladding. Carbon.

[14] Li S., Huang K., Zhang Z., Zheng C., Li M., Wang L., Wu K., Tan H., Yi X. (2023). Wear Mechanisms and Micro-Evaluation of WC + TiC Particle-Reinforced Ni-Based Composite Coatings Fabricated by Laser Cladding. Mater. Charact..

[15] Xiao Q., Sun W.L., Yang K.X., Xing X.F., Chen Z.H., Zhou H.N., Lu J. (2021). Wear Mechanisms and Micro-Evaluation on WC Particles Investigation of WC-Fe Composite Coatings Fabricated by Laser Cladding. Surf. Coat. Technol..

[16] Liu J., Yu H., Chen C., Weng F., Dai J. (2017). Research and Development Status of Laser Cladding on Magnesium Alloys: A Review. Opt. Lasers Eng..

[17] Shamsujjoha J., Ruano S.G., Siegfried J., Baker B., Thurston M., Walluk M., Holding R., Jonnalagadda A. (2025). Crack Mitigation and Wear Performance of High-Strength Steel Coatings Deposited by High-Speed Laser Cladding. Surf. Coat. Technol..

[18] Li X., Xiong J., Ou W., Yuan X., Zhang L., Xu J., Yang J., Jiao Y., Zhang H., Chen Z. (2023). Cracking Mechanisms and Prevention of Stellite 6# Surface Layer Built by Laser Cladding during Thermal Cyclic Process. Int. J. Fatigue.

[19] Fu R., Peng S., Di X., Guo Y., Liu C. (2026). Strong Interface Bonding in Copper/Steel Composites Enabled by High Cooling Rates during Twin-Wire Arc-Directed Energy Deposition. Mater. Sci. Eng. A.

[20] Chang Y.-Y., Qiu J.-R., Hwang S.-J. (2022). Multi-Objective Optimization of Directed Energy Deposition Process by Using Taguchi-Grey Relational Analysis. Int. J. Adv. Manuf. Technol..

[21] Deng D., Li T., Huang Z., Jiang H., Yang S., Zhang Y. (2022). Multi-Response Optimization of Laser Cladding for TiC Particle Reinforced Fe Matrix Composite Based on Taguchi Method and Grey Relational Analysis. Opt. Laser Technol..

[22] Wang M., Liu X., Jia H., Zhu Y., Wei Y., Shi T. (2025). Process Optimization, Interface Microstructure and Performance of Inconel 625 Coating on K4002 Nickel-Based Superalloy by inside-Laser Coaxial Powder Feeding Deposition. J. Alloys Compd..

[23] Shang A., Yang Q., Wang F., Wang S., Li D. (2026). Review of State-of-the-Art and Research Frontiers in Single-Crystal Turbine Blade Repair by Laser Directed Energy Deposition. Addit. Manuf. Front..

[24] Yang J., Huang J., Fan D., Chen S., Zhao X. (2016). LaAlO3 as the Heterogeneous Nucleus of Ferrite: Experimental Investigation and Theoretical Calculation. J. Alloys Compd..

[25] Zhang Z., Hu C.C., Chen H., He J. (2020). Pinning Effect of Reactive Elements on Structural Stability and Adhesive Strength of Environmental Sulfur Segregation on Al_2_O_3_/NiAl Interface. Scr. Mater..

[26] Gao W., Lian G., Liu J., Chen C., Feng M. (2025). CeO_2_-Induced Microstructural Refinement and Performance Optimization of Laser Cladding CoCrCu_0_._5_FeNiSi_2_ High-Entropy Alloy Coatings. J. Alloys Compd..

[27] Quazi M.M., Fazal M.A., Haseeb A.S.M.A., Yusof F., Masjuki H.H., Arslan A. (2016). Effect of Rare Earth Elements and Their Oxides on Tribo-Mechanical Performance of Laser Claddings: A Review. J. Rare Earths.

[28] Liu L., Wang G., Ren K., Di Y., Wang L., Rong Y., Wang H. (2022). Marangoni Flow Patterns of Molten Pools in Multi-Pass Laser Cladding with Added Nano-CeO_2_. Addit. Manuf..

[29] Feng Y., Feng K., Yao C., Li Z. (2019). Effect of LaB_6_ Addition on the Microstructure and Properties of (Ti_3_Al + TiB)/Ti Composites by Laser Cladding. Mater. Des..

[30] Chen C., Meiping W., Rui H., Yuling G., Xiaojin M. (2022). Understanding Stellite-6 Coating Prepared by Laser Cladding: Convection and Columnar-to-Equiaxed Transition. Opt. Laser Technol..

[31] Han W., Min J., Dai G., Guo Y., Chang L., Wang Y., Zhao E., Sun Z., Chang H. (2023). Effect of Y_2_O_3_ Addition on Microstructure and Properties of Ti_6_Al_4_V by Laser Melting Deposition. Mater. Sci. Eng. A.

[32] Zhang L., Zhang M., Zhu Z., Gao M., Gao J., Guo Z. (2022). Effects of Nano-CeO_2_ on Microstructure and Properties of Ni625 Alloy Prepared by Laser Cladding. J. Alloys Compd..

[33] Zhang B., Liu K., Li J., Chen B., Huang C.J., Soboleva N. (2025). A Comprehensive Review on the Rare Earth Elements Improving Microstructure and Properties of Laser Cladded Coatings. J. Alloys Compd..

[34] Wang L., Guo Q., Chen L., Yan W. (2023). In-Situ Experimental and High-Fidelity Modeling Tools to Advance Understanding of Metal Additive Manufacturing. Int. J. Mach. Tools Manuf..

[35] Siva Prasad H., Brueckner F., Kaplan A.F.H. (2020). Powder Incorporation and Spatter Formation in High Deposition Rate Blown Powder Directed Energy Deposition. Addit. Manuf..

[36] Liang C.J., Wang C.L., Zhang K.X., Liang M.L., Xie Y.G., Liu W.J., Yang J.J., Zhou S.F. (2021). Nucleation and Strengthening Mechanism of Laser Cladding Aluminum Alloy by Ni-Cr-B-Si Alloy Powder Based on Rare Earth Control. J. Mater. Process. Technol..

[37] Yao Q., Xu N., Wu X., Li T., Liu X., Zhu X., Jiao Y., Jiang Y. (2026). Atomic-Scale Characterization of Intermetallic Compounds in the La/Ce-Added Steel Rebars with High Corrosion Resistance. Mater. Charact..

[38] Sun C., Li W., Li C., Sun R., Liu G., Li X. (2025). Study on Microstructure and Fatigue Properties of Laser Powder Bed Fusion Nickel-Based Superalloy with Heat Treatment. Addit. Manuf. Front..

[39] Xue Q., Wang G., Di Y., Liu L., Shi W., Wang L. (2024). Modeling on Molten Pool Transport in Laser Deposition Processes by Lattice Boltzmann Method. Int. Commun. Heat Mass Transf..

[40] Wang W. (2022). Exploring the Nano-Polishing Mechanisms of Invar. Tribology International.

[41] Song W., He Q., Zhuo Y., Shao W., Zhang S., Ren X., Yang Q. (2023). First Principles Analysis on the Nucleation Interface of La_2_O_3_ (1 1 0) /NbC (1 1 0). Mater. Today Commun..

[42] He Y., Wang J., Zhu T., Ren X., Guo J., Yang Q. (2024). Insights into Refinement Mechanism of Primary M7C3 Carbide by La_2_O_3_ via Experiments and First-Principles Calculations. J. Mater. Res. Technol..

[43] Negami M., Morihashi R., Yoshino T., Sahara R., Yamabe-Mitarai Y. (2024). Effect of Reactive Elements in MCrAlX Bond Coat for Durability Improvement of Thermal Barrier Coatings. Corros. Sci..

[44] Ren X., Sun W., Sheng Z., Liu M., Hui H., Xiao Y. (2023). Effects of Nano-CeO_2_ on Microstructure and Properties of WC/FeCoNiCrMo0.2 Composite High Entropy Alloy Coatings by Laser Cladding. Nanomaterials.

[45] Wang C., Gao Y., Wang R., Wei D., Cai M., Fu Y. (2018). Microstructure of Laser-Clad Ni60 Cladding Layers Added with Different Amounts of Rare-Earth Oxides on 6063 Al Alloys. J. Alloys Compd..

[46] Liu Z., Miao X., Wu M., Wang H., Jie D., Zhang Y., Li J. (2025). Effect of Y_2_O_3_ on Microstructure and Tribology Properties of Laser Cladded WTaNbMo Refractory High-Entropy Alloy Coatings. Mater. Today Commun..

[47] Feng M., Lin T., Lian G., Chen C., Huang X. (2024). The Influence of WC Content on the Microstructure and Properties of Laser Cladding CoCrFeMnNiSi_1.6_ High-Entropy Alloy Coatings. Ceram. Int..

[48] Tang Y., Yang C.-P., Sun Q.-Q., Wu L.-K., Cao F.-H. (2024). Effects of TiC Particles on Tribological and Corrosion Resistance of PEO Coating on TC4 Alloy. Corros. Commun..

[49] Fu X., Zhang Y., Li J., Liu B., Zhang R., Fang Q. (2023). Uncertainty and Statistics of Dislocation-Oxide Interactions on Strength and Creep Failure of Oxide Dispersion Strengthened Steels. Eng. Fract. Mech..

[50] Peng S., Wang Z., Li J., Fang Q., Wei Y. (2023). Beyond Orowan Hardening: Mapping the Four Distinct Mechanisms Associated with Dislocation-Precipitate Interaction. Int. J. Plast..

[51] Du M., Wang L., Gao Z., Yang X., Liu T., Zhan X. (2022). Microstructure and Element Distribution Characteristics of Y_2_O_3_ Modulated WC Reinforced Coating on Invar Alloys by Laser Cladding. Opt. Laser Technol..

[52] Zheng C., Huang K., Mi T., Li M., Li S., Yi X. (2024). Impact of CeO_2_ Modification on the Quality and Wear Performance of Al_2_O_3_/SiC Reinforced Metal-Based Coatings. Mater. Charact..

[53] Xie Y., Jiang W., Xu K., Wen X., Huang B. (2025). Microhardness, Wear Resistance, and Corrosion Resistance of AlCoCrFeNi_2.1_+xCeO_2_ High-Entropy Alloy Coatings by Laser Cladding. Mater. Today Commun..

[54] Sahoo S.K., Ramesh M.R., Panigrahi S.K. (2025). Establishing High Temperature Tribological Performance and Wear Mechanism Map of Engineered In-Situ TiB2 Reinforced Mg-RE Metal Matrix Composites. Tribol. Int..

[55] Zhang H., Pan Y., Zhang Y., Lian G., Cao Q., Que L. (2022). Microstructure, Toughness, and Tribological Properties of Laser Cladded Mo2FeB2-Based Composite Coating with in Situ Synthesized WC and La2O3 Addition. Surf. Coat. Technol..

[56] Deng C., Chen W., Zhu Y. (2025). Experimental and Numerical Investigation of Bonding Strength in Laser Cladding: Fe-Based Cladding Layer with Varying WC Contents Coating on AISI H13 Substrate. Opt. Laser Technol..

[57] Chen Z., Yuan J., Wang M., Liu X., Sun Y., Wang H., Jiang K., Li C., Liu C., Wang Z. (2025). Comparative Study on Microstructure and Properties of WC / Cr_3_C_2_ Dual-Phase Enhanced Laser Cladding NiCrBSi Composite Coating. Mater. Today Commun..

[58] Weng Z., Wang A., Wu X., Wang Y., Yang Z. (2016). Wear Resistance of Diode Laser-Clad Ni/WC Composite Coatings at Different Temperatures. Surf. Coat. Technol..

[59] Zhu Y., Li C., Ye H., Li M., Hu C. (2025). Effect of Nanoscale CeO_2_ Powder on Wear and Corrosion Resistance of Ni60A-WC Coatings. Ceram. Int..

[60] Shu D., Dai S., Wang G., Si W., Xiao P., Cui X., Chen X. (2020). Influence of CeO2 Content on WC Morphology and Mechanical Properties of WC/Ni Matrix Composites Coating Prepared by Laser in-Situ Synthesis Method. J. Mater. Res. Technol..

[61] Zhang G., Liu W., Bian H., Xi W., Zao Y., Zhang K., Wang H. (2025). Influence Mechanisms of Y_2_O_3_ Addition on the Microstructure and Wear Resistance of Laser-Cladded T-800 + Si Coatings on DD5 Substrates. Surf. Coat. Technol..

[62] Wang J., Wang L., Chen C., Wang X., Zhao F. (2024). Effect of Rare Earth on Primary Carbides in H13 Die Steel and Their Addition Method: A Review. J. Iron Steel Res. Int..

[63] Tang Y., Qin L., Pan W., Yang Z., Li X. (2026). Threshold Effect and Synergistic Enhancement of Wear Resistance in CeO_2_-Modified Co-Based Coatings Fabricated via Low-Power Laser Cladding. Surf. Coat. Technol..

[64] Li J.R., Xie D.S., Zeng Z.R., Song B., Xie H.B., Pei R.S., Pan H.C., Ren Y.P., Qin G.W. (2023). Mechanistic Investigation on Ce Addition in Tuning Recrystallization Behavior and Mechanical Property of Mg Alloy. J. Mater. Sci. Technol..

[65] Wang J., Li J., Wang L., Chen C., Wang X., Zhao F. (2023). Study on Modification of Primary Carbides and Improvement of Properties in H13 Steel by CeO_2_ Nanoparticles Addition. J. Mater. Res. Technol..

[66] Zhang G., Hu T., Shuai S., Chen C., Xu S., Yu J., Ren W., Wang J., Ren Z. (2023). Ab-Initio Molecular Dynamics Study of Heterogeneous Nucleation at the Liquid-Y/α-Al_2_O_3_ Interface. Comput. Mater. Sci..

[67] Guo K., Sun Y., Cheng W. (2024). Effect of Rare Earth La2O3 on the Microstructure and Tribological Properties of Laser-Clad CoCrFeNiMoSi High-Entropy Alloy Coatings. Mater. Lett..

[68] Zhang H., Zou Y., Zou Z., Wu D. (2015). Microstructures and Properties of Low-Chromium High Corrosion-Resistant TiC–VC Reinforced Fe-Based Laser Cladding Layer. J. Alloys Compd..

[69] Zhang H., Zou Y., Zou Z., Shi C. (2014). Effects of CeO_2_ on Microstructure and Corrosion Resistance of TiC-VC Reinforced Fe-Based Laser Cladding Layers. J. Rare Earths.

[70] Ravi Kumar B., Sharma S., Munda P., Minz R.K. (2013). Structure and Microstructure Evolution of a Ternary Fe–Cr–Ni Alloy Akin to Super Martensitic Stainless Steel. Mater. Des..

[71] Chen X., Qiao G., Han X., Wang X., Xiao F., Liao B. (2014). Effects of Mo, Cr and Nb on Microstructure and Mechanical Properties of Heat Affected Zone for Nb-Bearing X80 Pipeline Steels. Mater. Des..

[72] Zhang H., Zou Y., Zou Z., Wu D. (2015). Microstructure and Properties of Fe-Based Composite Coating by Laser Cladding Fe–Ti–V–Cr–C–CeO_2_ Powder. Opt. Laser Technol..

[73] Farahmand P., Liu S., Zhang Z., Kovacevic R. (2014). Laser Cladding Assisted by Induction Heating of Ni–WC Composite Enhanced by Nano-WC and La_2_O_3_. Ceram. Int..

[74] Miao X., Wu M., Wang H., Zhao Y., Cui C., He R., Jie D. (2024). Effects of La Content on Microstructure and Tribological Properties of Laser Clad Ni60/WC/La_2_O_3_ Composite Coatings on Cr12MoV. J. Mater. Res. Technol..

[75] Cao L., Qian Y., Shao Y., Ren Y., Li Y., Cui H., Li Y., Wu Y. (2025). Microstructure and Properties of CeO_2_ and WC Synergistically Enhanced Laser Cladding Ni60 Coatings. J. Mater. Res. Technol..

[76] Gu D., Shen Y. (2007). Influence of La_2_O_3_ addition on the microstructure and forming properties of laser-sintered (WC-Co)p/Cu metal matrix composites. Acta Metall. Sin..

[77] Wang Q., Yang J., Niu W., Li Y., Mao X., Wang Y., Zhang K. (2021). Effect of La_2_O_3_ on Microstructure and Properties of Fe-Based Alloy Coatings by Laser Cladding. Optik.

[78] Ding L., Hu S. (2015). Effect of Nano-CeO_2_ on Microstructure and Wear Resistance of Co-Based Coatings. Surf. Coat. Technol..

[79] Liu Y., Deng C., Gong B., Bai Y. (2019). Effects of Heterogeneity and Coarse Secondary Phases on Mechanical Properties of 7050-T7451 Aluminum Alloy Friction Stir Welding Joint. Mater. Sci. Eng. A.

[80] Weng F., Yu H., Chen C., Liu J., Zhao L. (2015). Microstructures and Properties of TiN Reinforced Co-Based Composite Coatings Modified with Y_2_O_3_ by Laser Cladding on Ti–6Al–4V Alloy. J. Alloys Compd..

[81] Wu Y., Yan Q., Zhang X. (2020). Wear Characteristics of Fe-Based Diamond Composites with Cerium Oxide (CeO_2_) Reinforcements. Int. J. Refract. Met. Hard Mater..

[82] Zhang F.-Z., Liu X.-B., Chen G.-D., Cheng W., Zhou H.-B., Meng Y., Zhao H.-L., Zhu J.-P., Wang K.-M. (2026). Tribo-Corrosion Performance of Laser Cladding CuNiTiCrNb(h-BN)x High-Entropy Alloy Coatings. Tribol. Int..

[83] Wang K., Wang Z., Peng P., Wu X., Liu W., Zhang H., Zhang H., Li Z., Ju J. (2026). Synergistic Enhancement of Strength and Wear Resistance in Nickel-Based Superalloy Fabricated by Oscillating Laser Directed Energy Deposition via Laser Shock Peening. J. Mater. Res. Technol..

[84] He R., Wu M., Jie D., Cui C., Ou B., Miao X., Gong Y. (2023). A Novel Approach to Regulate the Microstructure of Laser-Clad FeCrNiMnAl High Entropy Alloy via CeO_2_ Nanoparticles. Surf. Coat. Technol..

[85] Liu X., Du Y., Pei X., Wang H., Hua D., Zhou Q., Wang H. (2025). Unlocking Metallic Glasses Ultra-Low Friction via High-Entropy Effect and Oxidation. Int. J. Mech. Sci..

[86] Ju J., Wang X., Xiao B., Zhu H., Tran N., Shen Z., Zeng X., Wang J., Sun B., Liaw P.K. (2026). Eliminating Intergranular Oxidation and Microstructural Instability in Chemically Complex Intermetallic Alloys Featuring Nano-Disorder Interfaces. Corros. Sci..

[87] Gamanov Š., Holzer J., Roupcová P., Svoboda J. (2022). High Temperature Oxidation Kinetics of Fe-10Al-4Cr-4Y_2_O_3_ ODS Alloy at 1200–1400 °C. Corros. Sci..

[88] Wang K., Yi Z., Song Z., Hu Y., Pang X., Zhang J., Ji X., Fang J., Wu P., Zhang M. (2025). Wear Behaviors of Laser-Directed Energy Deposited Inconel 625 Alloys-Based Composite Coating by Different Oscillating Laser Paths. Opt. Laser Technol..

[89] Wang Z.-H., Liu X.-B., Zhang C.-Y., Zhong R., Zou K., Deng Q.-Y., Wang K.-M., Gu J. (2026). Study of Tribological and Salt Spray Corrosion Properties of Laser-Cladded Ni60-Ti_2_AlC Composite Coatings on 316 L Stainless Steel. Tribol. Int..

[90] Wang M., Hua D., Li J., Jiao Z., Zhu L., Xie M., Zhu Y., Huang T., Ye W., Lin N. (2025). Interaction between Dislocations and L12 Precipitates in the Al0.3CoCrFeNi High-Entropy Alloy during Nanoscratching. Tribol. Int..

[91] Fan Z., Wang K., Dong X., Wang R., Duan W., Mei X., Wang W., Cui J., Zhang S., Xu C. (2017). The Role of the Surface Morphology and Segmented Cracks on the Damage Forms of Laser Re-Melted Thermal Barrier Coatings in Presence of a Molten Salt (Na_2_SO_4_+V_2_O_5_). Corros. Sci..

[92] Tian H., Wang X., Cui Z., Lu Q., Wang L., Lei L., Li Y., Zhang D. (2018). Electrochemical Corrosion, Hydrogen Permeation and Stress Corrosion Cracking Behavior of E690 Steel in Thiosulfate-Containing Artificial Seawater. Corros. Sci..

[93] Michael Joseph Stalin P., Arjunan T.V., Matheswaran M.M., Manoj Kumar P., Sadanandam N. (2021). Investigations on Thermal Properties of CeO_2_/Water Nanofluids for Heat Transfer Applications. Mater. Today Proc..

[94] Wang J., Kong L., Li T., Xiong T. (2016). A Novel TiAl_3_/Al_2_O_3_ Composite Coating on γ-TiAl Alloy and Evaluating the Oxidation Performance. Appl. Surf. Sci..

[95] Du X., Ma X., Ding X., Zhang W., He Y. (2022). Enhanced High-Temperature Oxidation Resistance of Low-Cost Fe–Cr–Ni Medium Entropy Alloy by Ce-Adulterated. J. Mater. Res. Technol..

[96] Xia D., Song S., Zhu R., Behnamian Y., Shen C., Wang J., Luo J., Lu Y., Klimas S. (2013). A Mechanistic Study on Thiosulfate-Enhanced Passivity Degradation of Alloy 800 in Chloride Solutions. Electrochim. Acta.

[97] Xi X., Lin D., He Z., Ma R., Wei H., Shi Z., Zhao W., Chen B., Tan C., Dong Z. (2025). Additively Manufactured Crack-Free Nickel-Based Superalloy with Synergistic Strength and Plasticity via Grain Refinement and Striped Oxides Inhibition. Compos. Part B Eng..

[98] Li Z., Zhao W., Yu K., Guo N., Xiao G., Wang Z., Zhang H. (2024). Effect of Y_2_O_3_ on Microstructure and Properties of CoCrFeNiTiNb High Entropy Alloy Coating on Ti–6Al–4V Surface by Laser Cladding. J. Rare Earths.

[99] Cui T., Leng W., Zhou D., Yu M., Yuan Z., Li J., Wang Y., Wang L. (2021). Improved Hot Corrosion Resistance of Al-Gradient NiSiAlY Coatings at 750 °C by Pre-Oxidation. Surf. Coat. Technol..

[100] Mannava V., Rao A.S., Paulose N., Kamaraj M., Kottada R.S. (2016). Hot Corrosion Studies on Ni-Base Superalloy at 650 °C under Marine-like Environment Conditions Using Three Salt Mixture (Na_2_SO_4_+NaCl+NaVO_3_). Corros. Sci..

[101] Ju J., Yu H., Kong H., Qi D., Peng P., Shen Z., Ding C., Ma S., Gao H., Wang J. (2025). Unveiling Oxidation Mechanism and Microstructural Evolution of L-DED Ni3Al-Based Intermetallic Alloy at 800 °C via Multi-Scale Characterization and Thermodynamic Calculations. Nano Mater. Sci..

[102] Lin F., Wang Z., Xu X., Luo K., Lu J. (2024). The Effect of Y_2_O_3_ Nanoparticle Addition on the Microstructure and High-Temperature Corrosion Resistance of IN718 Deposited by Extreme High-Speed Laser Cladding. Surf. Coat. Technol..

[103] Qu M., Guo Q., Escano L.I., Nabaa A., Hojjatzadeh S.M.H., Young Z.A., Chen L. (2022). Controlling Process Instability for Defect Lean Metal Additive Manufacturing. Nat. Commun..

[104] Cunningham R., Zhao C., Parab N., Kantzos C., Pauza J., Fezzaa K., Sun T., Rollett A.D. (2019). Keyhole Threshold and Morphology in Laser Melting Revealed by Ultrahigh-Speed x-Ray Imaging. Science.

[105] Sun J., Wen Y., Wang Z., Zhang J., Wang L., Qu X., Zhang B. (2023). Effect of Lithium Anti-Ablation and Grain Refinement Introduced by TiC Nanoparticles in LPBF Al–Li Alloy. J. Mater. Res. Technol..

[106] Jia D., Shi W., Li K., Lu C., An F., Lin L., Guo F. (2024). Effects of Rare Earth Oxides on Wear Resistance and Corrosion Resistance of 316L/TiC Composite Coating by Laser Cladding. Mater. Today Commun..

[107] Cai J., Wei J., Zu Z., Guan Q., Lyu P. (2022). Comparative Hot Corrosion Performance of Arc Ion Plated NiCoCrAlYSiHf Coating in Na_2_SO_4_/NaCl/V_2_O_5_-Media via High-Current Pulsed Electron Beam. Mater. Charact..

[108] Fan J., Chen C., Yao H., Xu K., Liu J., Pu J. (2025). Corrosion and Wear Behaviors of AlCoCr_1_+Fe_1_-Ni_2_.1 High-Entropy Alloys. Corros. Commun..

[109] Huang X.-H., Liu X.-B., Cheng W., Zhong R., Ren X.-P., Deng Q.-Y., Wang K.-M. (2026). Regulating the High-Temperature Tribological Performance and Oxidation Behavior of High-Entropy Composite Coating via Ti_3_SiC_2_: Long-Term Service of TC4 Alloy. Tribol. Int..

[110] Li S., Liu D., Liu G., Xin S., Deng Z., Li C., Chen T. (2023). Study on the Preparation of CeO2-Doped AlCoCrFeNiTi High Entropy Alloy Bioinert Coatings by Laser Cladding: The Effect of CeO_2_ Particle Size on the Tribological Properties of the Coatings. Surf. Coat. Technol..

[111] Iqbal A., Moskal G., Cavaleiro A., Amjad A., Khan M.J. (2024). The Current Advancement of Zirconate Based Dual Phase System in Thermal Barrier Coatings (TBCs): New Modes of the Failures: Understanding and Investigations. Alex. Eng. J..

[112] Yuling G., Meiping W., Xiaojin M., Chen C. (2021). Effect of CeO_2_ on Crack Sensitivity and Tribological Properties of Ni60A Coatings Prepared by Laser Cladding. Adv. Mech. Eng..

[113] Habibi M.H., Wang L., Liang J., Guo S.M. (2013). An Investigation on Hot Corrosion Behavior of YSZ-Ta2O5 in Na2SO4+V2O5 Salt at 1100 °C. Corros. Sci..

[114] Habibi M.H., Wang L., Guo S.M. (2012). Evolution of Hot Corrosion Resistance of YSZ, Gd_2_Zr_2_O_7_, and Gd_2_Zr_2_O_7_+YSZ Composite Thermal Barrier Coatings in Na_2_SO_4_+V_2_O_5_ at 1050 °C. J. Eur. Ceram. Soc..

[115] Guo L., Zhang C., Li M., Sun W., Zhang Z., Ye F. (2017). Hot Corrosion Evaluation of Gd_2_O_3_-Yb_2_O_3_ Co-Doped Y_2_O_3_ Stabilized ZrO_2_ Thermal Barrier Oxides Exposed to Na_2_SO_4_+V_2_O_5_ Molten Salt. Ceram. Int..

[116] Wang J., Sun J., Zou B., Zhou X., Dong S., Li L., Jiang J., Deng L., Cao X. (2017). Hot Corrosion Behaviour of Nanostructured Zirconia in Molten NaVO_3_ Salt. Ceram. Int..

[117] Iqbal A., Moskal G. (2023). Recent Development in Advance Ceramic Materials and Understanding the Mechanisms of Thermal Barrier Coatings Degradation. Arch. Comput. Methods Eng..

[118] Shen Z., Liu G., Huang B., Dai J., Mu R., He L. (2022). Effect of Er on Thermal Property and Failure Behaviour of LaErZrO Thermal Barrier Coatings. Corros. Sci..

[119] Zhen Z., Wang X., Shen Z., Mu R., He L., Xu Z. (2021). Thermal Cycling Behavior of EB-PVD Rare Earth Oxides Co-Doping ZrO_2_-Based Thermal Barrier Coatings. Ceram. Int..

[120] Li W., Yang Y., Han K., Sun S., Zhou F. (2024). Study on the Microstructure and Wear Resistance of Fe-Co-Sm Alloy Coatings with Different Sm Contents Assisted by Alternating Magnetic Field during Laser Cladding. Mater. Today Commun..

[121] Abdel Gawad A.E. (2021). Mineral Chemistry (U, Th, Zr, REE) in Accessory Minerals from Wadi Rod Elsayalla Granitoids, South Eastern Desert, Egypt. Arab. J. Geosci..

[122] Cabral Pinto M., Silva M., Neiva A., Guimarães F., Silva P. (2018). Release, Migration, Sorption, and (Re)Precipitation of U during Peraluminous Granite Alteration under Oxidizing Conditions in Central Portugal. Geosciences.

[123] Lin S., Zhang T. (2025). Review of Rare Earth Oxide (Nano and Micro Sized Crystalline) Materials: Preparation from Rare Earth Chlorides, Characterisation and Applications. Hydrometallurgy.

[124] Dagwar P.P., Iqbal S.S., Dutta D. (2025). Sustainable Recovery of Rare Earth Elements from Industrial Waste: A Path to Circular Economy and Environmental Health. Waste Manag. Bull..

